# The YTHDF proteins modulate Alzheimer’s disease-associated brain gene signatures

**DOI:** 10.1186/s13024-026-00986-6

**Published:** 2026-09-05

**Authors:** Shinya Tasaki, Denis R. Avey, Nicola A. Kearns, Chunjiang Yu, Sashini De Tissera, Himanshu Vyas, Lin Cheng, Jishu Xu, Artemis Iatrou, Daniel J. Flood, Wenlong Li, Lisa L. Barnes, Katherine Rothamel, Aliza P. Wingo, Thomas S. Wingo, Nicholas T. Seyfried, Chuan He, Philip L. De Jager, Gene W. Yeo, Chris Gaiteri, David A. Bennett, Yanling Wang

**Affiliations:** 1 Rush Alzheimer’s Disease Center, Rush University Medical Center, Chicago, IL 60612 USA; 2 Department of Psychiatry, McLean Hospital, Harvard Medical School, Belmont, MA 02478 USA; 3 Department of Chemistry, Department of Biochemistry and Molecular Biology, Institute for Biophysical Dynamics, Howard Hughes Medical Institute, University of Chicago, Chicago, IL USA; 4 Center for RNA Technologies and Therapeutics, Department of Cellular and Molecular Medicine, University of California San Diego, La Jolla, CA 92093 USA; 5 Department of Psychiatry, University of California, Davis, CA 95817 USA; 6 VA Northern California Health Care System, Sacramento, CA 95652 USA; 7 Department of Neurology, University of California, Davis, CA 95817 USA; 8 Department of Biochemistry, Emory University School of Medicine, Atlanta, GA 30322 USA; 9 Department of Neurology and the Taub Institute for the Study of Alzheimer’s Disease and the Aging Brain, Center for Translational and Computational Neuroimmunology, Columbia University Medical Center, New York, NY 10032 USA; 10 Department of Psychiatry, Upstate Medical University, Syracuse, NY 13210 USA

**Keywords:** Alzheimer's disease, Cognitive decline, Gene expression signatures, Machine learning, Post-transcriptional regulation, Epitranscriptomics, eCLIP-seq, CRISPR knockout, Small-molecule YTHDF inhibition, Cortical organoids

## Abstract

Gene signatures of Alzheimer’s disease (AD) brains reflect the output of a complex interplay of genetic, epigenetic, epi-transcriptomic, and post-transcriptional regulations. To nominate candidate factors modulating these signatures, we developed a machine learning model to integrate cellular and molecular features explaining differential gene expression in AD. Among the features tested, YTHDF proteins, the canonical readers of N6-methyladenosine (m6A) RNA modification, are among the most influential predictors of AD gene signatures. Protein modules containing YTHDFs were downregulated in human AD brains, and knockdown or pharmacological inhibition of YTHDFs in iPSC-derived 2D and 3D neuronal models recapitulated key AD-associated gene signatures. Furthermore, eCLIP-seq revealed altered YTHDF binding to transcripts in AD brains, at both m6A-dependent and m6A-independent sites. Together, these results support an important role for YTHDF proteins in modulating AD-associated gene signatures in the human brain.

**Supplementary Information:**

The online version contains supplementary material available at https://doi.org/10.1186/s13024-026-00986-6.

## Main

Many RNA expression changes have been identified in Alzheimer’s disease (AD) brains [1–3]. These changes could be influenced by multiple factors, such as genetic, epigenetic, epi-transcriptomic, and post-transcriptional mechanisms. These regulatory factors often interact in a cell-type-specific manner, contributing to the complexity and heterogeneity of the disease process [4]. However, it remains unclear which of these factors is primarily responsible for driving the AD gene signature and, ultimately, how to halt the progression of molecular dysregulation in AD. Existing purely data-driven approaches, such as co-expression networks [1–3], are valuable but can be difficult to link to specific genomic mechanisms. In contrast, knowledge-based approaches, such as genomic interaction databases (e.g., transcription factor-DNA, RNA-RNA, protein-RNA interactions) generated by consortia [5–7] hold valuable insights; however, their integration with gene expression data has often been insufficient for defining overarching disease mechanisms. These challenges have recently motivated the application of explainable machine learning approaches [8] to efficiently integrate multi-omics data, identify expression patterns, and predict top regulators and disease mechanisms.

In this study, we integrate these two approaches by discovering regulatory features of gene signatures associated with cognitive decline in AD using our DEcode-tree model. This model predicts that YTH domain-containing family proteins (YTHDFs), the classical reader proteins of N6-methyladenosine (m6A) RNA modification, shape the gene signatures of AD brains. We validate this prediction in human brains by global proteomics, and test it directly by knocking down and pharmacologically inhibiting YTHDFs in iPSC-derived 2D and 3D neuronal models, which recapitulate neuronal components of AD gene signatures. We then perform enhanced crosslinking and immunoprecipitation followed by sequencing (eCLIP-seq) to map YTHDF binding sites and m6A sites in the same brains, revealing altered YTHDF–transcript binding in AD relative to cognitively normal brains at both m6A-dependent and m6A-independent sites. Together, these results support an important role for YTHDF proteins in shaping AD gene signatures and nominate them as candidate therapeutic targets. Collectively, our study highlights the power of integrating machine learning, multi-omics data, and gene perturbation to predict, identify, and validate critical regulators of AD gene signatures.

## Results

### m6A readers are among the most influential predictors for differentially expressed genes in AD

**Fig. 1:**
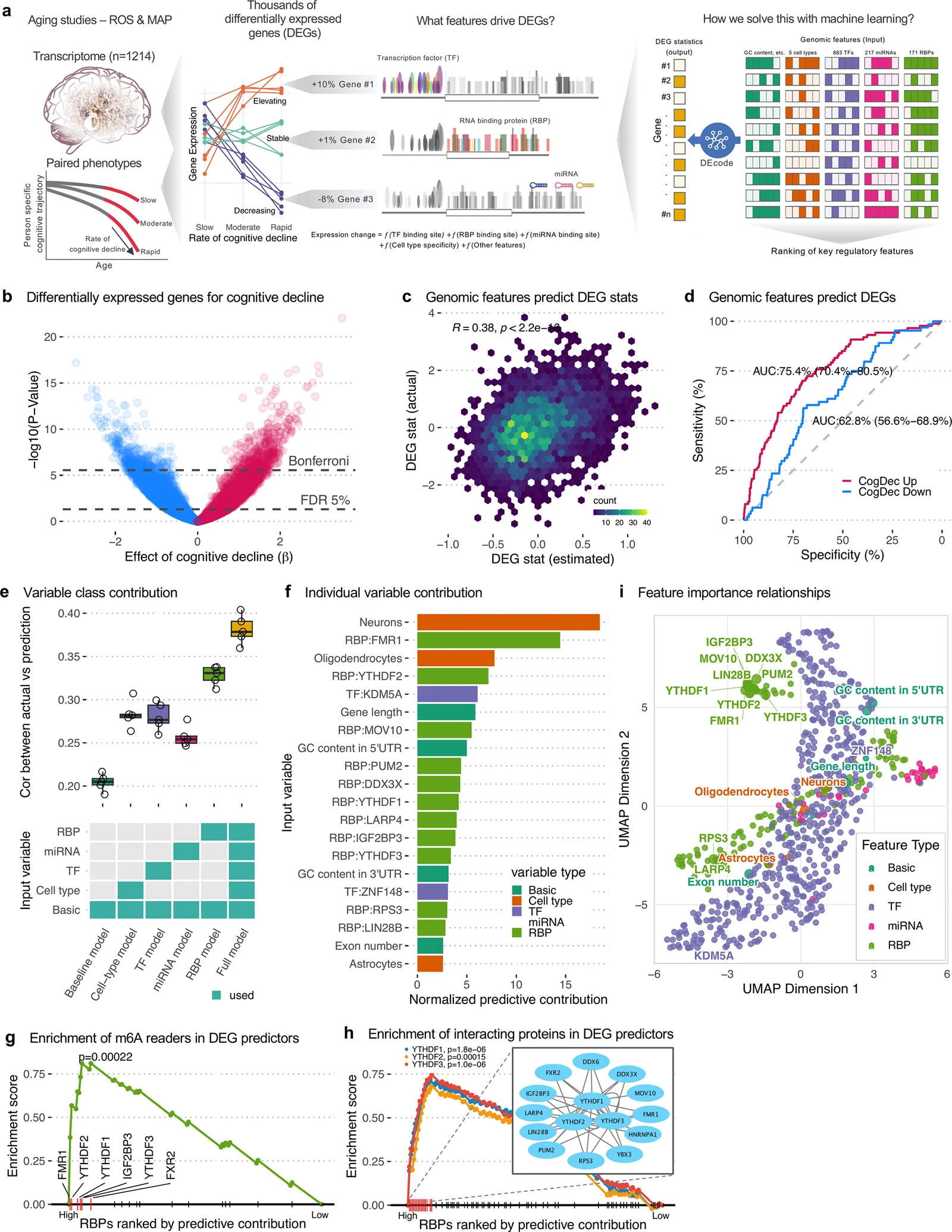
Decode-tree model predicts top regulatory features of differentially expressed genes associated with cognitive decline in DLPFC. (**a**) Predicting key regulatory factors for gene signature of cognitive decline. We applied DEcode-tree to ROSMAP brain transcriptome data and predicted genes associated with cognitive decline. We then factorized the origin of differential expression into several types of gene regulatory information, including basic gene info (number of exons, 5‘UTR length, transcript length, GC content), binding sites (transcription factors, RBPs, and miRnas), and gene expression profiles of five types of neural cells (neurons, astrocytes, microglia, oligodendrocytes, and oligodendrocyte progenitor cells). (**b**) Volcano plot showing differentially expressed genes for cognitive decline. Differential expression analysis was based on DLPFC RNA-seq data from 761 ROSMAP participants, with person-specific cognitive decline rates computed from yearly performance tests. (**c**) Based on gene features, the DEcode-tree predicts differential gene expression statistics (t-stat) and (**d**) differentially expressed genes in dementia. Pearson’s correlation and *p*-value are shown in the two-dimensional density plot. The ROC curve provides binary classification accuracy, with confidence intervals computed via bootstrapping. (**e**) RBPs as key predictors of differential expression in dementia. Variable importance was assessed via ablation analysis, repeated five times with different training and test gene sets, shown as dots on the boxplot. (**f**) Ranking of influential variables based on SHAP scores. The top 20 variables, ranked by absolute SHAP score, are shown with colors indicating their respective classes. (**g**) m6A readers are enriched among the model’s influential variables. Gene-set enrichment analysis of known m6A readers was conducted against the ranking of RBPs based on their predictive contribution. (**h**) Proteins interacting with YTHDF1-3 are enriched among the model’s influential variables. Gene-set enrichment analysis of proteins interacting with YTHDF1-3 was conducted against the ranking of RBPs based on their predictive contribution. (**i**) UMAP projection of feature contributions in differential gene expression prediction. Two-dimensional representation of feature correlations derived from SHAP value matrices in a DEcode-tree model predicting gene expression changes associated with cognitive decline. The 20 most influential features are labeled. Proximity in UMAP space reflects similarity in how features contribute to predicting gene expression changes

Transcriptome analysis of bulk tissue from large-scale brain cohorts has identified many differentially expressed genes (DEGs) associated with AD phenotypes, such as neuropathologies and cognitive decline [1] (Fig. 1a, b). In the ROSMAP cohort, these AD-associated transcriptional signatures are unlikely to be primarily explained by changes in cellular composition. RNA-seq deconvolution revealed only modest differences in cell-type proportions across diagnostic groups, and adjustment for estimated cell-type proportions preserved the majority of AD-associated DEGs (Supplementary Fig. 1a, b). Since these gene signatures potentially reflect the integral output of multifaceted regulatory mechanisms, it remains challenging to untangle this complexity, uncover among the most influential mechanisms shaping these specific gene signatures, and identify their cell types of action.

To address this challenge, we built a machine learning framework, DEcode-tree, that explains DEGs related to the rate of cognitive decline in AD. With interpretability as our foundation, we compiled biophysical features of basic gene information (number of exons, 5’ UTR length, transcript length, GC content) and regulatory features of binding sites (833 transcription factors, 172 RNA-binding proteins, and 217 miRNAs), along with gene expression profiles of major neural cell types (neurons, astrocytes, microglia, oligodendrocytes, and oligodendrocyte precursor cells). Through the integration of these diverse data types, DEcode-tree captures the multifaceted regulatory landscape influencing gene expression changes associated with cognitive decline. Crucially, DEcode-tree predicts differential-expression status for genes never seen during training using a gene-holdout paradigm, enabling discovery of transferable regulatory principles rather than memorizing gene-specific patterns. We applied DEcode-tree to the DLPFC RNA-seq data from 761 ROSMAP participants (Supplementary Table 1). By contrasting gene expression levels with the rate of cognitive decline, we identified 6,855 DEGs at an FDR of 5%, and 775 DEGs after Bonferroni correction (Fig. 1b, and Supplementary Table 2). These DEG statistics remained highly concordant after adjustment for estimated cell-type proportions (*R* = 0.96; Supplementary Fig. 1c). Our model predicted DEGs with an overall correlation of *R* = 0.38 and *p* = 6.66×10^−120^ to the actual DEG statistics (Fig. 1c). The AUC (Area Under the Curve) value was 0.75 (90% CI: 0.70–0.81) for upregulated genes and 0.63 (90% CI: 0.57–0.69) for downregulated genes (Fig. 1d). DEcode-tree achieved a 47% improvement in predictive performance over our previous DEcode method [8] (Supplementary Fig. 2b). DEcode-tree achieved an overall correlation of *R* = 0.38, representing approximately 60% of the empirical maximum observed when comparing t-statistics between independent batches of expression data (R ~ 0.63, Supplementary Fig. 2c). This empirical ceiling reflects the inherent biological variability and technical noise in gene expression measurements, positioning our model’s performance as robust within the constraints of gene regulatory complexity.

To identify the most impactful features in our model, we conducted an ablation analysis by systematically removing each input, retraining the model, and then evaluating the model’s performance. Notably, the model trained with RNA-binding protein (RBP) binding sites performed better (*R* = 0.33, median absolute deviation (MAD) = 0.01) than models trained with other inputs such as transcription factor (TF) binding sites (*R* = 0.28, MAD = 0.03), in predicting the cognitive decline gene signature (Fig. 1e), highlighting the importance of post-transcriptional regulation via RBPs in AD pathophysiology.

To narrow down the essential individual features in our model, we computed the SHAP score [9], a value that represents the contribution of each feature to the prediction. We found that the most predictive cell types were neurons, oligodendrocytes, and astrocytes (Fig. 1f). Aligning with our ablation analysis, the RBPs are the essential predictors (Fig. 1f and Supplementary Table 3), and among the RBPs, the canonical reader proteins for m6A modification, including FMR1 and YTHDF1, YTHDF2, and YTHDF3, were among the most influential predictors (Fig. 1f). These RBPs are preferentially bound to up-regulated differentially expressed genes, with approximately 75% of upregulated genes bound by YTHDF1 and YTHDF2, and slightly lower but significant binding percentages observed for YTHDF3 and FMR1 (Supplementary Fig. 2d). Because m6A is one of the most common RNA modifications and plays a crucial role in regulating RNA stability, translation, and degradation, our finding prompted us to examine the performance of all m6A reader proteins in our model. To do that, we conducted gene-set enrichment analysis using 23 known m6A readers [10] and detected their significant enrichment among all RBP predictors (*p* = 2.2 × 10^−4^) (Fig. 1g). Given that YTHDFs often interact with other proteins to regulate RNA metabolism [11], we then calculated the enrichment of their interacting proteins among the RBP predictors. We found that the interacting proteins of YTHDF1 (*p* = 1.8 × 10^−6^), YTHDF2 (*p* = 1.5 × 10^−4^), and YTHDF3 (*p* = 1.0 × 10^−6^) were also enriched (Fig. 1h). To further understand the relationships between predictors in our model, we analyzed pairwise SHAP correlations using UMAP visualization (Fig. 1i). This analysis revealed that m6A-related RBPs form a distinct, tightly clustered group, confirming their coordinated contribution to differential expression and supporting their known protein-protein interactions. These findings further underscore the importance of m6A-related RBPs in regulating gene expression related to cognitive decline in AD.

To further confirm the impact of RBPs on the model’s performance in predicting DEGs associated with cognitive decline, we trained the model on an independent batch of transcriptome data (*n* = 453). We achieved highly congruent results (Supplementary Fig. 2e). Furthermore, we applied the DEcode-tree to predict DEGs associated with AD diagnosis (Supplementary Table 2) and observed a similarly strong predictive power of RBPs and m6A reader proteins (Supplementary Fig. 2f). Collectively, our findings suggest that post-transcriptional regulation via RBPs, especially m6A readers, shapes AD molecular phenotypes.

### YTHDF-containing protein modules explain more of the AD gene signature than comparable modules

**Fig. 2:**
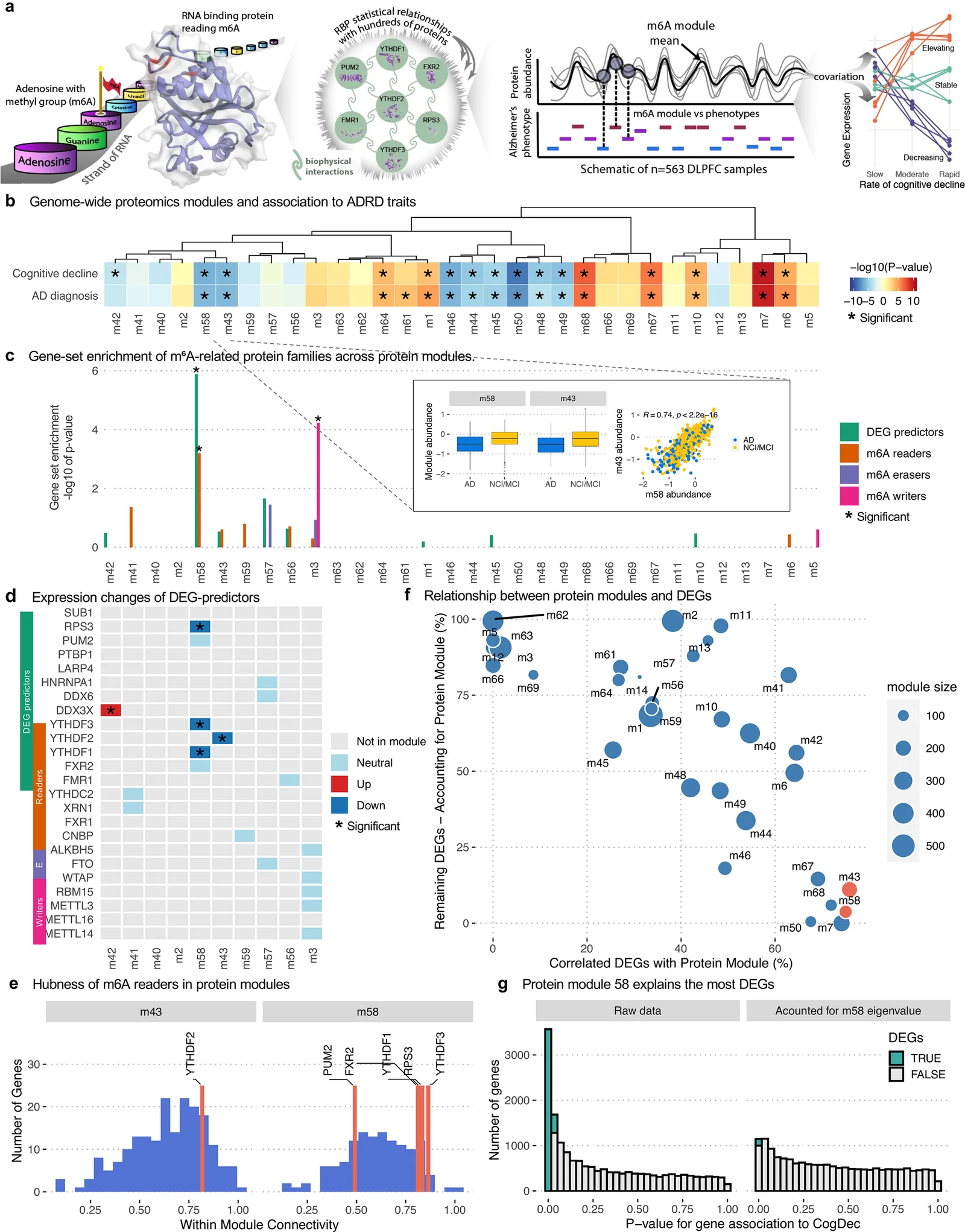
The m6A reader-containing modules explain the majority of AD differentially expressed genes. (**a**) Overview of the integrative analysis of proteomic data from 596 ROSMAP individuals, 549 of whom also have paired transcriptome data. (**b**) Protein co-abundance modules generated using SpeakEasy and their associations with cognitive decline and AD diagnosis. A dendrogram shows the relationships between modules based on the correlation of module eigenvalues. A heatmap displays the association of module eigenvalues with cognitive decline and AD diagnosis, using a linear regression model. Red indicates increased module abundance in individuals with rapid cognitive decline or AD, while blue indicates a decrease. An asterisk denotes statistical significance at an FDR of 5%. (**c**) Enrichment of DEG predictors, m6A readers, m6A erasers, and m6A writers in each protein module. Notably, m58 is enriched for DEG predictors and known m6A reader proteins, whereas m3 is enriched for m6A writers. However, m58 is associated with cognitive decline and AD diagnosis, while m3 is not. Enrichment was tested using Fisher’s exact test, and an asterisk indicates statistical significance at an FDR of 5%. An inset shows that m58 and m43 are highly correlated (*R* = 0.74; *p* < 2.2e-16), and their DLPFC expression is lower in AD compared to individuals with no cognitive impairment (NCI) or mild cognitive impairment (MCI). (**d**) At the individual protein level, RPS3 and YTHDFs decrease in AD individuals, and these proteins reside in m58 and m43. An asterisk indicates statistical significance at nominal *p* < 0.05, as determined by a linear regression model. (**e**) m6A reader proteins display more intra-module connections with other proteins within m43 or m58. Connectivity within the module for all genes is represented as a histogram, with the connectivity values for m6A readers shown by red vertical bars. (**f**) Correlation values of protein modules with the percentage of DEGs (x-axis) and the percentage of remaining DEGs after accounting for the module effect (y-axis). The eigenvalues of m58 and m43 are significantly correlated with more than 70% of DEGs in AD dementia (x-axis) but are correlated with only 5–10% after accounting for their module effects (y-axis). (**g**) An example of how accounting for m58 affects the *p*-value distribution in DEG analysis for cognitive decline

Based on the observation that RNA targets of m6A reader RBPs are differentially expressed in AD, we next examined the dysregulation of the RBPs themselves. Since RBP transcripts must be translated into proteins, to subsequently bind RNA, we analyzed the protein levels of m6A-related RBPs in AD DLPFC using global proteomics data from 596 ROSMAP individuals (549 of which had paired transcriptome data) (Supplementary Table 4). As RBPs often interact with other proteins to mediate various post-transcriptional processes, we first constructed protein co-abundance modules from 7,640 detected global proteins (Supplementary Table 5), and then associated the average expression of each module with rapid cognitive decline and AD clinical diagnosis (Fig. 2a). Among the 33 protein modules (range: 35 to 567 proteins; median: 194), 16 modules were significantly associated with cognitive decline, 16 modules were associated with AD diagnosis (FDR at 5%), and 15 modules were associated with both traits (Fig. 2b and Supplementary Table 6). To provide broader molecular context for RBPs, we examined which modules contained m6A-related proteins and the key DEG predictors identified by DEcode-tree (Fig. 1f and Supplementary Table 3). m6A writers were enriched in module m3 (463 proteins, *p* = 6.0 × 10^−5^), but this module was not associated with either trait. Notably, we found that DEG predictors (*p* = 1.3 × 10^−6^) and cytoplasmic m6A readers (*p* = 6.2 × 10^−4^) were both significantly enriched in m58 (125 proteins) (Fig. 2c and Supplementary Table 6), and associated with cognitive decline and AD diagnosis. Moreover, the sets of interacted proteins of YTHDF1 (*p* = 6.5 × 10^−11^), YTHDF2 (*p* = 5.0 × 10^−14^), and YTHDF3 (*p* = 1.2 × 10^−7^) were all enriched in m58 (Supplementary Table 6).

At the individual protein level, the expression of RPS3, YTHDF1, and YTHDF3 in m58, and YTHDF2 in m43 (194 proteins), was reduced in AD individuals with rapid cognitive decline (Fig. 2d). Confirming these results we replicated in an independent TMT proteomics dataset (*n* = 288), and meta-analysis across both datasets confirmed significant downregulation of these RBPs (RPS3: *p* = 4.4×10^−9^, YTHDF1: *p* = 1.5 × 10^− 4^, YTHDF2: *p* = 7.6 × 10^− 4^, YTHDF3: *p* = 2.9 × 10^− 4^) and their containing modules (m58: *p* = 1.0 × 10^−14^, m43: *p* = 4.6 × 10^−12^) (Supplementary Fig. 3a). Notably, m58 and m43 were highly correlated with each other, and they were negatively associated with AD and cognitive decline (Fig. 2b, c). We did not detect those m6A reader changes at the RNA level and the preservation of the m58 and m43 modules at the RNA level (Supplementary Fig. 3b, c). This observation aligns with our previous study, which showed a generally poor correlation between RNA and protein levels and their covariance structures in human brains [12]. To evaluate whether these m6A readers connect with other proteins in the same module, we calculated the strength of intra-module connectivity of each protein within m58 and m43. Indeed, the m6A readers demonstrated high connectivity with other proteins in their respective modules, indicating their “hub” function (Fig. 2e).

This led us to hypothesize that if m6A readers control the AD gene signature, m58 and m43 expression would account for the DEGs associated with cognitive decline (Fig. 2a). To test this hypothesis, we leveraged the paired proteomic and transcriptome data from 549 DLPFC samples. Specifically, we computed two key metrics: the percentage of DEGs significantly correlated with protein module levels, and the percentage of DEGs remaining after accounting for module effects. Both metrics revealed a substantial impact of m58 and m43 on the DEGs associated with cognitive decline (Fig. 2f and Supplementary Table 6). The initial set of 3,971 DEGs was reduced to 149 genes after accounting for the effects of m58 (Fig. 2g), and similar results were observed for m43 (Supplementary Fig. 3d). Because any protein module strongly associated with cognitive decline would be expected to absorb part of this transcriptomic variance, we next asked whether the YTHDF-containing modules explained a disproportionate fraction of the DEG signature. To test this, we modeled the percentage of DEGs explained across all 33 protein modules using a quasibinomial generalized linear model, adjusting for module size and strength of trait association and treating m58 and m43 as a pre-specified class based on their coordinated network organization. The YTHDF-containing modules explained significantly more DEGs than expected under this matched null (OR = 3.25, *p* = 0.016; Supplementary Fig. 3e). Although a substantial fraction of the DEG reduction is shared with other strongly AD-associated modules, these results indicate that the m6A reader modules account for more of the cognitive decline gene signature than expected based on their size and disease association.

Taken together, we show that m6A readers and their broader protein modules are negatively associated with cognitive decline and AD diagnosis. These m6A readers interact broadly with other proteins within the same module. Accounting for the effect of m6A reader modules offsets the most of gene expression changes related to cognitive decline. Together with the matched-null analysis, these results are consistent with the predictions of our DEcode-tree model and show that m6A-related RBPs and their protein modules explain more of the AD brain gene signature than other protein modules of similar size and disease association.

### Genetic perturbations of m6A-related RBPs recapitulate the AD-associated gene signatures in iPSC-derived cortical cells

Consistent transcriptomic and proteomic evidence indicates that m6A-related RBPs are involved in shaping AD gene signatures. However, whether they play a causal role or are merely correlates remains unclear. Because m6A readers were downregulated in AD brains (Fig. 2d), we reasoned that reducing their expression in human cells would help define their functional consequences. To test this, we used a commercially available neural progenitor cell (NPC) line derived from standard iPSC cortical differentiation, representing an early cortical progenitor stage as confirmed by established marker expression [13–15].

**Fig. 3:**
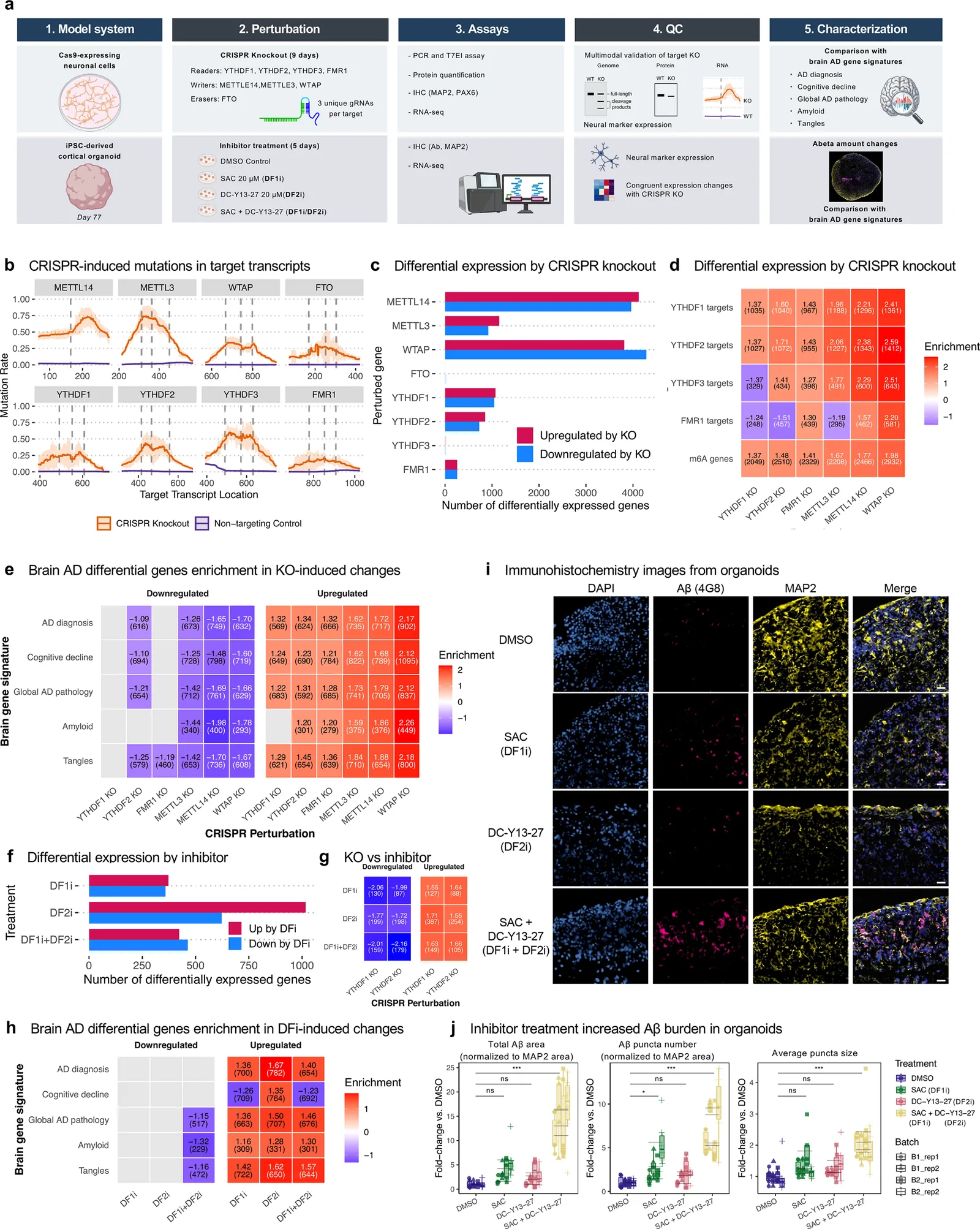
Perturbation of m6A-associated RBPs induces AD-like transcriptomic and pathological phenotypes. (**a**) Flowchart illustrating the experimental and analytical overview of the CRISPR/Cas9 perturbation and pharmacological inhibition experiments. (**b**) Mutation rates induced by the perturbations along the targeted regions. Mutation rates were calculated from RNA-seq reads, with dashed lines indicating the target transcript locations of gRnas. (**c**) Number of differentially expressed genes induced by each perturbation. Differentially expressed genes were identified with an FDR threshold of 5%. (**d**) Enrichment of RBP target genes and genes with known m6A modifications in CRISPR-perturbed transcriptional changes. Gene set enrichment analysis was performed using the fgsea package in **R.** Colored cells indicate statistical significance at FDR 5%; grey cells indicate non-significance. (**e**) Enrichment of AD brain gene signatures in CRISPR-perturbed transcriptional changes. Gene set enrichment analysis was performed using gene rankings based on t-statistics from CRISPR perturbation and AD brain signatures as gene sets. Colored cells indicate statistical significance at FDR 5%; grey cells indicate non-significance. (**f**) Number of differentially expressed genes induced by each inhibitor treatment. Differentially expressed genes were identified with an FDR threshold of 5%. (**g**) Enrichment of inhibitor-induced DEGs in CRISPR KO transcriptional changes. Gene set enrichment analysis was performed using inhibitor-induced DEGs as gene sets. Colored cells indicate statistical significance at FDR 5%; grey cells indicate non-significance. (**h**) Enrichment of AD brain gene signatures in inhibitor-induced transcriptional changes. Gene set enrichment analysis was performed using gene rankings based on t-statistics upon inhibitor treatment and AD brain signatures as gene sets. Colored cells indicate statistical significance at FDR 5%; grey cells indicate non-significance. (**i**) Representative immunohistochemistry images from organoids treated with inhibitors or DMSO. Sections were stained for DAPI, Aβ, and MAP2 (scale bars: 20 microns). (**j**) Inhibitor treatment increased Aβ accumulation. Boxplots showing comparison of total Aβ area, Aβ puncta number, and Aβ puncta size across DMSO, DF1i, DF2i, and DF1i+DF2i conditions (*p < 0.05, ***p < 1e-5, ns: not significant)

To enable CRISPR/Cas9 editing, we transduced NPCs with Cas9-expressing lentiviruses and expanded the cultures for two weeks (Supplementary Fig. 4a). This strategy introduced CRISPR-Cas9 machinery while allowing the cells to mature toward a late progenitor stage. We then cultured the Cas9-expressing cells for an additional 6 days before transfecting them with synthetic guide RNAs (gRNAs) targeting major m6A readers (YTHDF1, YTHDF2, YTHDF3, and FMR1), writers (METTL14, METTL3, and WTAP), or the eraser FTO. After a further 9-day perturbation period, the cultures exhibited the expected heterogeneity of cortical differentiation, comprising PAX6^+^ progenitor cells and MAP2^+^ neurons (Supplementary Fig. 4b). We harvested both RBP-targeting and non-targeting control (NTC) cultures for RNA-seq and knockdown-validation experiments. Editing efficiency was confirmed at the DNA level using a T7 endonuclease I assay, and knockdown efficiency was further verified using western blotting and RNA-seq–derived transcript abundance (Fig. 3b and Supplementary Fig. 4c, d).

We next conducted differential gene expression analysis on the RNA-seq data to characterize KO-induced transcriptional changes. This analysis revealed hundreds to thousands of DEGs for each perturbation, with the exception of YTHDF3 and FTO (Fig. 3c and Supplementary Table 7). Notably, although YTHDF3 emerged as an important predictor in the DEcode-tree model, its individual perturbation resulted in relatively few DEGs. This observation suggests that predictive importance in the computational model does not necessarily correspond to the magnitude of the transcriptional response following perturbation of an individual regulator.

To determine whether these KOs affect genes bound by m6A readers or genes harboring m6A modifications, we performed gene-set enrichment analysis. We found that these KOs significantly upregulated corresponding target genes and genes with known m6A modifications, consistent with these RBPs functioning as post-transcriptional suppressors (Fig. 3d). We next examined whether the KO-induced DEGs overlapped with five AD-associated gene signatures derived from postmortem DLPFC tissue, specifically those linked to AD diagnosis, cognitive decline, global AD pathology, amyloid burden, and tau pathology. Across all five AD-associated gene signatures, we observed a strong and consistent positive correlation, whereby KO-induced upregulated genes correspond to AD-upregulated signatures, and KO-induced downregulated genes mapped to AD-downregulated signatures (Fig. 3e and Supplementary Table 8). These results show that reducing YTHDFs activity recapitulates key AD-associated transcriptional features in cultured iPSC-derived cortical cells.

### Pharmacological inhibitions of YTHDF proteins recapitulate AD-related gene signatures and Aβ pathology in iPSC-derived cortical organoids

To validate the CRISPR-based perturbation findings in an orthogonal 3D human neural model, we generated cortical organoids from iPSC lines derived from cognitively normal ROSMAP participants using established differentiation protocols [16]. Day-77 organoids were treated with SAC (YTHDF1 inhibitor) [17], DC-Y13-27 (YTHDF2 inhibitor) [18], or both inhibitors in combination, followed by multimodal characterization five days later, including RNA-seq and immunostaining (Fig. 3a). To confirm the expected 3D organization, we stained organoids for PAX6/TBR2 (progenitor zones) and TBR1/CTIP2/SATB2 (neuronal layers) (Supplementary Fig. 4e).

RNA-seq analysis revealed hundreds of DEGs across all inhibitor conditions (Fig. 3f and Supplementary Table 9). Notably, transcriptomic changes induced by inhibitor treatment were strongly concordant with those observed in the corresponding CRISPR KOs (Fig. 3g), indicating on-target engagement of YTHDF1/2. We next evaluated whether inhibitor-induced DEGs aligned with the same five AD-related transcriptomic signatures. Across all signatures, AD-upregulated genes showed strong and consistent enrichment among inhibitor-upregulated genes, whereas enrichment of AD-downregulated genes was more limited and primarily detectable in the DF1+DF2 inhibition condition (Fig. 3h). Thus, pharmacological inhibition preferentially recapitulates the AD-upregulated gene program.

Because Aβ predominantly accumulates within neuronal regions, Aβ measurements were normalized to MAP2+ area to account for regional and organoid-to-organoid variability. Organoids treated with either SAC or DC-Y13-27 alone showed minimal changes in total Aβ area, Aβ puncta number, or average puncta size, whereas combined inhibition of YTHDF1 and YTHDF2 led to marked increases across all Aβ metrics compared with DMSO controls (Fig. 3i–j;) Importantly, neuronal integrity, quantified as the MAP2-positive area normalized to DAPI area, was not significantly different among treatment groups (Supplementary Fig. 4f-g). In addition, no coordinated activation of apoptosis, TP53 signaling, the integrated stress response (ISR), the unfolded protein response (UPR), or heat-shock pathways was observed following inhibitor treatment (Supplementary Fig. 5), supporting that the enhanced Aβ phenotype is unlikely to be attributable to generalized neuronal stress.

Together, genetic and pharmacological perturbations studies support a role for reduced YTHDF activity in modulating AD-associated transcriptomic signatures and AD-like neuropathological phenotypes in human iPSC-derived neuronal models.

### Partial overlapping of m6A reader binding sites with m6A-modification sites in postmortem DLPFC

**Fig. 4:**
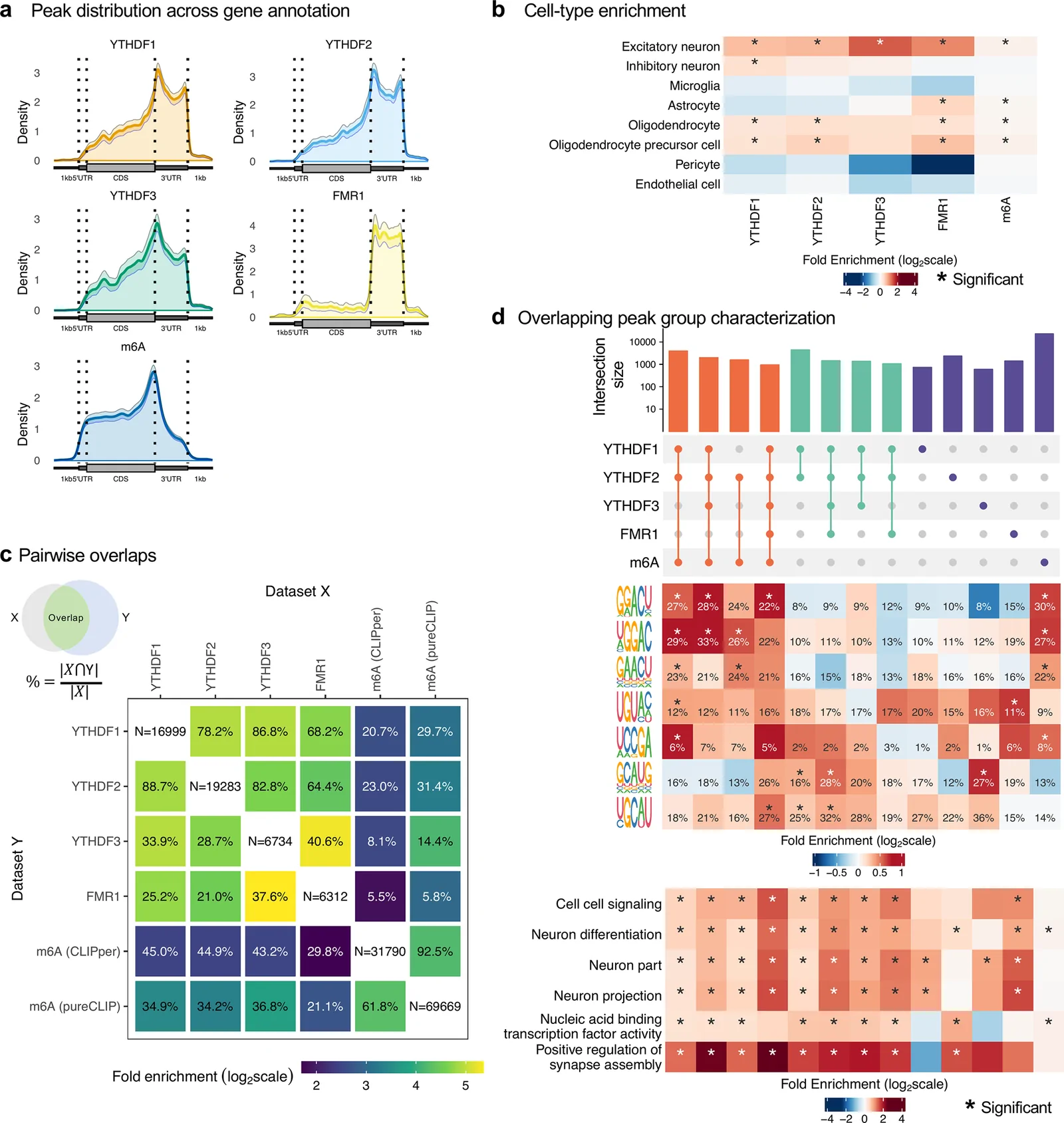
Comprehensive analysis of m6A reader bindings and m6A modifications in postmortem brains. (**a**) Distribution of detected peaks across annotated transcript regions for YTHDFs, FMR1, and m6A, based on eCLIP experiments on DLPFC samples from 39 AD and 42 non-cognitively impaired (NCI) individuals. (**b**) Cell type enrichment of genes bound by m6A reader proteins or containing m6A modifications. Gene sets with eCLIP peaks were compared against gene sets preferentially expressed in brain cell types. Statistical significance was assessed using Fisher’s exact test, with an asterisk indicating significance at an FDR of 5%. (**c**) Overlap analysis between m6A modification sites and reader binding sites. The ratio of overlapping peaks between antibodies was calculated, and Fold enrichment was assessed using the binomial test. There is significant overlap between m6A readers and m6A sites (3.26- to 17.9-Fold), and a much stronger overlap between m6A readers themselves (21.1- to 40.8-Fold). (**d**) Stratification of eCLIP peak groups based on overlapping patterns into three major groups: peak group 1, with colocalized peaks for m6A and YTHDF1, YTHDF2, YTHDF3, or FMR1; peak group 2, with colocalized peaks for RBPs only; and peak group 3, with no colocalized peaks (top). Motif analysis shows both canonical and non-canonical m6A/A motifs, which are significantly enriched in peak group 1, but to a lesser extent in peak groups 2 and 3 (middle). Gene ontology analysis indicates that genes associated with canonical or non-canonical pathways are strongly linked to synaptic signaling assembly and neuronal projections (bottom). Motif enrichment analysis for each peak group was conducted using monaLisa. An asterisk indicates significance at a nominal *p*-value < 0.0001, as determined by Fisher’s exact test

In order to examine whether m6A readers recognize m6A-modified transcripts in postmortem human brains, we conducted enhanced crosslinking and immunoprecipitation (eCLIP) to profile transcriptome-wide binding sites for YTHDF1, YTHDF2, YTHDF3, and FMR1, along with m6A-modified sites on DLPFC samples from 39 AD and 42 non-cognitively impaired (NCI) individuals (Supplementary Table 10). First, we performed QC analysis to assess the variability across sequencing batches and between samples to ensure the consistency and reliability of the data (Supplementary Fig. 6a, b). We then used the peak-calling tool CLIPper to identify peaks and determined consensus peaks, i.e., peaks present in at least 50% of samples, for YTHDF1, YTHDF2, YTHDF3, FMR1, and m6A (Supplementary Fig. 6c and Supplementary Table 11). Consistent with previous reports [19, 20], peaks for YTHDF1, YTHDF2, YTHDF3, and FMR1 were primarily located both at the boundary between coding sequences (CDS) and 3′ untranslated regions (3′ UTRs) as well as within the 3′ UTRs, whereas m6A peaks were primarily found at the CDS-3′ UTR boundary (Fig. 4a).

To understand in which cell type (s) the m6A modifications and YTHDF binding activities occur, we tested whether genes containing m6A reader and m6A peaks were enriched among marker genes of major brain cell types. We found that the genes with m6A reader and m6A peaks were primarily enriched in excitatory neurons; genes with YTHDF1, YTHDF2, FMR1, and m6A peaks were also enriched in oligodendrocytes and oligodendrocyte precursor cells; genes with m6A and FMR1 peaks were also enriched in astrocytes (Fig. 4b).

It is well known that m6A readers recognize m6A sites and mediate downstream RNA metabolism [21]. We reasoned that for this canonical pathway, we would expect to observe overlapping peaks between m6A and m6A readers. Conversely, a lack of overlapping peaks between m6A and m6A readers would suggest a non-canonical m6A-independent pathway. To this end, we first examined the peak overlaps for any given pair among m6A readers and m6A. As expected, we detected a significant overlap between m6A readers and m6A (3.26- to 17.9-fold). In addition, we also observed a much stronger overlap among m6A readers (21.1- to 40.8-fold) (Fig. 4c). However, the maximum observed overlap between m6A reader peaks and m6A peaks was 45%, suggesting that m6A readers can bind to RNAs independently of m6A sites.

To further examine sequence dependency, we conducted motif analysis on YTHDFs and m6A peaks. As expected, we found that the GGACU motif, a common DRACH sequence where the m6A modifications occur on RNA, was enriched in the peaks for YTHDF1, YTHDF2, YTHDF3, and m6A (Supplementary Fig. 7a). Notably, we also detected non-canonical motifs, where different nucleotides flanked the A/m6A for reader and m6A peaks (Supplementary Fig. 7a). We then clustered all peaks into three major groups based on the peak colocalization pattern: Peak Group 1 with colocalized peaks for m6A and YTHDF1, YTHDF2, YTHDF3, or FMR1; Peak Group 2 with colocalized peaks for RBPs only; and Peak Group 3 with no colocalized peaks (singletons) (Fig. 4d and Supplementary Table 11). Interestingly, both canonical and non-canonical m6A/A motifs were significantly enriched in Peak Group 1, but to a lesser extent in Peak Groups 2 and 3 (Fig. 4d and Supplementary Fig. 7b). Despite these differences, the genes bound by canonical or non-canonical pathways largely overlap. Specifically, of the 3,927 genes with peaks in Group 1 and the 2,846 genes in Group 2, 2,325 genes (81.7%) overlapped. This considerable overlap suggests that the co-occurrence of both peak types within the same gene is a prevalent feature. Reflecting this observation, both groups were strongly associated with synaptic signaling assembly, indicating their convergent functional effects (Fig. 4d).

For additional confirmation from a different methodology, we also used pureCLIP to identify m6A modification sites at a single-nucleotide resolution and detected similar peak distribution and motif enrichment to those revealed by CLIPper (Supplementary Fig. 7c, d). Taken together, our results suggest that the m6A readers may regulate gene expression post-transcriptionally via both m6A-dependent canonical and m6A-independent non-canonical pathways [22]. Our findings suggest a broader scope for the gene regulatory potential of m6A reader proteins.

### Altered m6A modifications and m6A-reader binding activities in AD brains

**Fig. 5:**
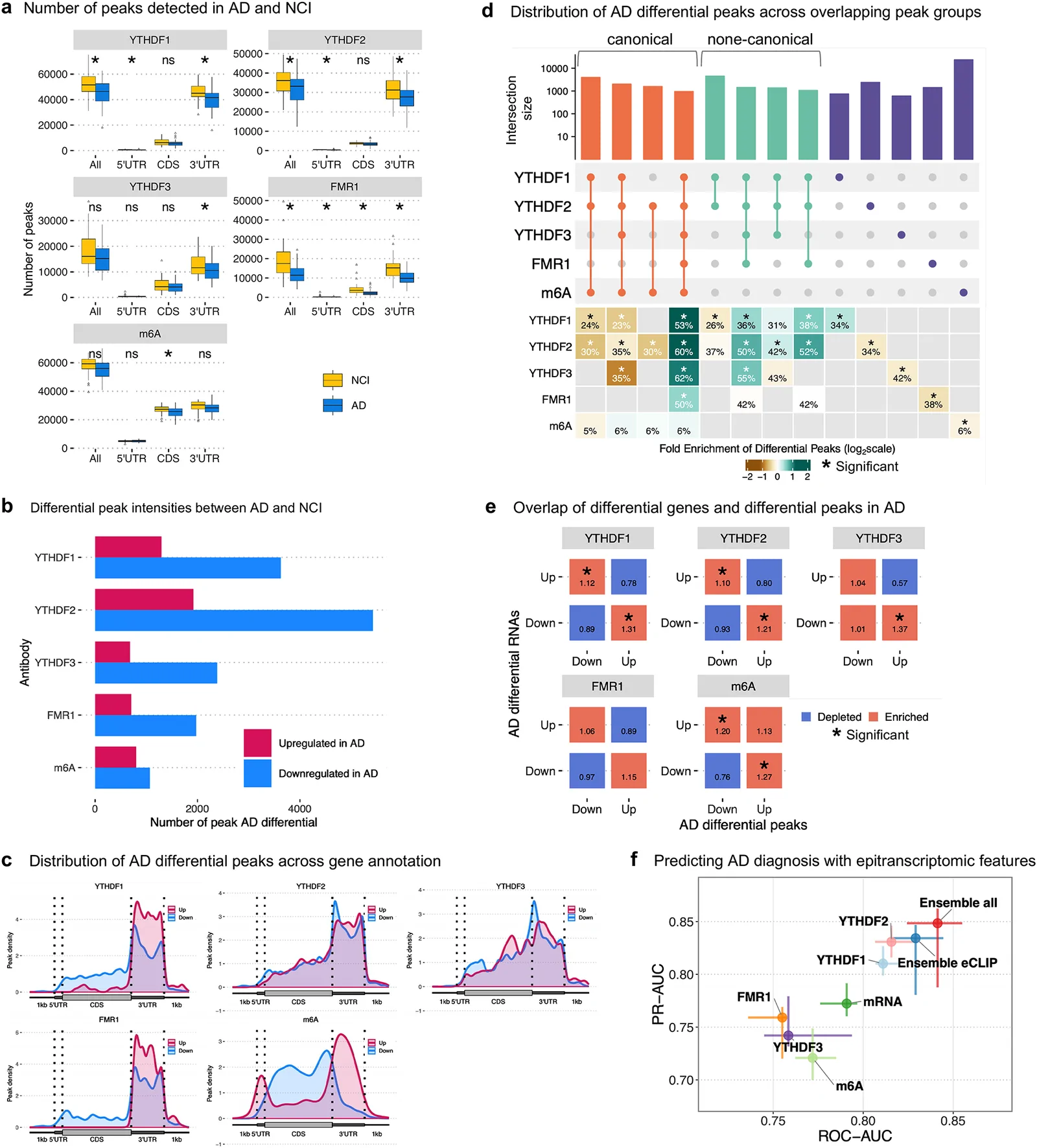
Differential m6A-reader bindings and m6A modifications in AD brains. (**a**) The overall number of peaks for m6A and m6A RBPs in NCI and AD, based on the presence and absence of peaks for any given location of transcriptome. Statistical significance was assessed using the non-parametric Wilcoxon test. An asterisk denotes a nominal *p*-value of <0.05. (**b**) The number of AD differential peaks for m6A and m6A RBPs based on a normalized peak intensity. Differential peak expression analysis was performed based on the negative binomial distribution. The bar plot indicates peaks passing a false discovery rate (FDR) threshold of 5%. (**c**) Distribution of AD differential peaks across annotated transcript regions for m6A and RBPs (**d**) Enrichment of AD differential peaks for m6A readers and m6A in each peak group. Statistical significance was determined using Fisher’s exact test. An asterisk indicates significance at an FDR of 5%. (**e**) Overlap of AD DEGs and AD differential peaks reveals an anti-correlation relationship for all m6A readers. Upregulated DEGs were associated with decreased peak intensities, whereas downregulated DEGs were associated with increased peak intensities. Statistical significance was determined by Fisher’s exact test. (**f**) Predictive models developed for AD diagnosis using eCLIP and/or RNA-seq data. Notably, YTHDF2 demonstrates superior accuracy in predicting AD compared to other m6A readers, with its predictive power approaching that of an ensemble model combining eCLIP and transcriptomic data

We investigated differential peaks for m6A modification and YTHDF binding activities in AD versus NCI brains. First, we took a binary approach to call the presence or absence of peaks for any given location in the transcriptome, and then calculated the overall peak numbers in NCI and AD brains. After adjusting for sequencing depth, batch, and RNA integrity, we detected a general reduction of peaks for all reader proteins and m6A in AD brains (Fig. 5a). Interestingly, the reduction in peaks for m6A reader proteins occurred primarily in 3’ UTRs, whereas the reduction in peaks for m6A was in CDS (Fig. 5a). Along with the reduced expression of YTHDFs and YTHDF-containing protein modules in AD brains (Fig. 2b, c, d), our results support a reduced trend of m6A modification and m6A reader binding activities in AD brains, which is largely in line with the results from previous animal and human studies [23–25].

For robustness, we also used a graded approach to gain a deeper understanding of differential m6A reader binding activities and m6A modifications in AD. To achieve this, we counted read numbers spanning each consensus peak and then normalized the reads to the parental RNA expression to derive a normalized peak intensity. This graded approach enabled transcriptome-wide peak association analysis, identifying peaks with significant differential intensity in AD. We detected more up- and down-regulated differential peak intensities in m6A reader proteins than in m6A modifications (Fig. 5b and Supplementary Table 12). Interestingly, the up- and down-regulated peaks for m6A reader proteins were primarily located in the 3’UTR, consistent with their typical peak distribution pattern (Figs. 4a and 5c). Conversely, the up-regulated m6A peaks were found in the 5’ UTR and 3’ UTR, whereas down-regulated m6A peaks were in the CDS, which deviated from the normal peak distribution pattern of m6A (Figs. 4a and 5c). This pattern shift of m6A differential peak intensity may reflect broader RNA processing changes in response to AD pathogenic processes. Building on our previous clustering of peaks into three major groups based on their colocalization patterns (Fig. 4d), where Peak Group 1 represents the m6A-dependent canonical pathway and Peak Groups 2 and 3 represent the m6A-independent non-canonical pathway, we sought to examine the enrichment of AD differential peaks within these groups. Differential peak enrichment was observed in both m6A-dependent and independent pathways. Specifically, AD differential peaks were positively enriched in regions colocalized with all RBPs—YTHDF1, YTHDF2, YTHDF3, and FMR1—and m6A within Peak Groups 1 and most of Peak Groups 2 (Fig. 5d). Conversely, AD differential peaks were depleted in m6A-dependent pathways lacking FMR1 and in singleton peaks, with the exception of YTHDF1. These findings suggest that the m6A-independent non-canonical pathway may also play a prominent role in regulating AD gene signatures.

To examine whether the changes in m6A modification and m6A reader binding activity in AD brains are associated with transcriptomic changes, we mapped peak changes to individual DEGs. We detected an inverse correlation for all m6A readers, i.e., upregulated DEGs were associated with decreased peak intensities, whereas downregulated DEGs were associated with increased peak intensities (Fig. 5e). This inverse relationship is exemplified by genes showing significant differential expression and opposite-direction changes in m6A reader binding across multiple reader proteins (Supplementary Fig. 7e). We note, however, that peak intensity is normalized to parental RNA expression, such that the inverse relationship in Fig. 5e cannot be fully separated from a potential normalization-dependent effect and should be interpreted as descriptive rather than mechanistic. Given the significant overlap of peaks between reader proteins (Fig. 4c), this consistent relationship across all reader proteins indicates that these proteins may work on a shared set of transcripts. Finally, we developed predictive models for AD diagnosis using eCLIP and/or RNA-seq data (Fig. 5f). Notably, YTHDF2 demonstrated superior accuracy in predicting AD compared to other m6A readers, with its predictive power close to an ensemble model combining eCLIP and transcriptomic data (Fig. 5f). Along with our proteomics and gene perturbation results, our findings indicate that YTHDFs, especially YTHDF2, are significantly associated with AD pathology and provide valuable insights into the molecular mechanisms underlying gene expression changes in AD brains.

## Discussion

In this study, we uncover a novel mechanism underlying molecular changes in AD, highlighting an important role for m6A reader proteins in modulating AD-associated gene signatures. Utilizing DEcode-tree, a machine learning model, we identify m6A-related RBPs as key regulators of gene expression changes in AD. Importantly, we then validate our predictions across multiple independent approaches, including proteomic analysis, eCLIP-seq experiments, and in vitro perturbations. Specifically, we demonstrate that YTHDFs and YTHDF-containing protein modules are downregulated in AD; ablation of YTHDF-containing modules in silico accounts for the majority of differentially expressed genes in AD; reducing m6A-related RBP activity in iPSC-derived neuronal cells and 3D organoids recapitulates the AD-associated gene signatures and Aβ-associated neuropathological phenotypes; m6A reader binding activities and m6A modifications are altered in AD brains. Our findings support a role for m6A readers in modulating AD-associated transcriptomic changes and provide evidence that perturbation of YTHDF activity recapitulates key molecular and pathological features observed in AD.

While our DEcode-tree model achieved an overall correlation of *R* = 0.38 with differentially expressed genes (Fig. 1c), this modest correlation reflects inherent limitations in predicting complex transcriptional changes in Alzheimer’s disease. Subsampling analyses between independent RNA-seq batches revealed that correlation plateaus at approximately *R* ≈ 0.63 even with large sample sizes, establishing an empirical upper bound constrained by biological variability and technical noise (Supplementary Fig. 1b). Our model’s performance thus captures approximately 60% of the empirically attainable signal. Importantly, despite this modest overall correlation, the ranking of key regulatory features remains highly consistent across independent datasets (Spearman *r* = 0.77), with m6A readers consistently emerging among the top predictors (Supplementary Fig. 1d). This consistency, coupled with our orthogonal experimental validations through proteomics, CRISPR perturbations, and eCLIP experiments, provides strong evidence for the biological significance of our findings. Additionally, prediction accuracy is fundamentally constrained by the limited availability of comprehensive RNA-protein interaction data [8], as global RNA-binding profiles exist for only approximately 10% of known RBPs, affecting all gene regulation-based models in this domain.

Numerous studies have demonstrated that m6A pathways play essential roles in neural development, synaptic plasticity, stress response, learning, and memory [26–28]. Emerging evidence also reveals that disruption of m6A pathways contributes to neurodegeneration and cognitive decline. For instance, the levels of m6A writers, m6A readers, and global m6A modifications are altered in AD mouse models and human AD brains [23–25, 29–31]. Furthermore, reduced neuronal m6A modifications lead to significant memory deficits, extensive synaptic loss, and neuronal death in mouse models [23, 24]. The m6A readers, such as YTHDF1 and YTHDF2, have been shown to play essential roles in learning and memory in animal models by regulating local protein synthesis and controlling mRNA localization [20, 32, 33]. Our study expands upon previous research by providing evidence that m6A reader proteins play an important role in modulating AD gene signatures. Using AI-based integrative analysis of human brain multi-omics data, along with gene perturbation experiments in human neuronal cells, we identify YTHDF proteins as important modulators of AD-associated gene signatures. This integrative and unbiased approach presents a robust framework for identifying, in silico testing, and experimentally validating gene regulators.

To examine whether human genetic evidence provides independent support for the involvement of YTHDF proteins in AD, we analyzed the most recent multi-ancestry Alzheimer’s disease GWAS meta-analysis (111,326 cases and 677,663 controls) [34]. We observed a nominally significant gene-level association for YTHDF1 (Cauchy *p* = 0.0097), while YTHDF2 and YTHDF3 showed no association (*p* ≈ 0.80 and 0.70, respectively). FMR1, located on the X chromosome, lacks available GWAS summary statistics. However, FXR2, an autosomal homolog of FMR1 that shares functional domains and is also highlighted by our DEcode-tree model (Fig. 1g) and involved in the protein module 58, shows significant association (Cauchy *p* = 0.0019). Loss-of-function constraint metrics from gnomAD [35] indicate that these genes are depleted of predicted loss-of-function variation relative to expectation, though to differing degrees: LOEUF = 0.132 for YTHDF2, 0.272 for YTHDF3, and 0.321 for FXR2, all below the conventional < 0.35 threshold for strong constraint, whereas YTHDF1 (LOEUF = 0.515) falls above this threshold while still remaining below the genome-wide median (~1.0). The strong constraint on YTHDF2 and YTHDF3 may explain their lack of GWAS signal. Variation that substantially disrupts genes under this degree of selective constraint tends to be removed from the population and therefore remains rare, whereas GWAS is powered to detect common variants. For such genes, a null association carries limited information about whether they contribute to disease biology. Although YTHDF2 and YTHDF3 do not exhibit significant associations in current AD GWAS, they emerged consistently across our computational modeling, human brain molecular profiling, and functional perturbation analyses, supporting their potential involvement in AD-associated molecular alterations through post-transcriptional regulatory mechanisms.

These results provide a holistic view of the m6A epi-transcriptomic landscape of the aging brain, through comprehensive epi-transcriptomic profiling of m6A modifications and m6A reader binding in human brains. The functions of YTHDFs in regulating RNA metabolism have been controversial. The prevailing model suggests that each YTHDF protein has distinct functions by binding to specific mRNAs, e.g., YTHDF1 enhances mRNA translation, while YTHDF2 mediates mRNA decay [21]. Conversely, the competing model proposes that YTHDF proteins have redundant functions, binding identically to all m6A sites on mRNAs and together mediating mRNA degradation in HeLa cells [36]. Furthermore, recent evidence supports a context-dependent model where YTHDFs interact with various partner proteins and RNAs within cytosolic condensates, such as stress granules [22, 37–39]. Our findings align with both the competing model and the context-dependent model for the following reasons. First, we observed significant overlap among m6A reader binding sites, either m6A-dependent or m6A-independent, indicating that different m6A reader proteins can bind the same sites on the same transcript; alternatively, different transcripts of the same gene can have the same sites recognized by different readers. Second, the association between reader binding and AD-associated differential expression was directionally consistent across readers, rather than reader-specific or opposing, suggesting a model of shared target recognition. We also detected both canonical, m6A-dependent motifs (Peak Group 1) and non-canonical m6A-independent motifs (Peak Group 2) (Fig. 5d). This suggests that reader proteins may bind directly to non-canonical sequences, regardless of m6A modification, or indirectly via protein-protein interactions. Genes bound via both canonical and non-canonical pathways are strongly associated with synaptic signaling and neuronal projection (Fig. 4d), indicating their convergent cellular functions. In summary, our results reveal the complexity of YTHDF proteins in gene regulation, which may allow for fine-tuned, context-dependent gene regulations in AD development.

Although we employed an integrative framework for gene regulator nomination and validation using extensive multi-omics datasets, several limitations should be acknowledged. First, the ROSMAP cohort primarily consists of White individuals, which may limit the generalizability of our findings to other ancestral populations. Second, although this represents one of the largest eCLIP datasets currently available, the sample size remains insufficient to extend the analysis beyond a case-control design. Future studies with substantially larger cohorts will be required to investigate disease stage-specific regulatory changes and evaluate the specificity of YTHDF dysregulation across AD and other neurodegenerative disorders. Third, differential reader binding was quantified by normalizing IP read counts to parental transcript expression. While this normalization distinguishes changes in reader occupancy from changes in transcript abundance, it also makes the resulting metric dependent on parental expression. Thus, the inverse relationship between differential reader binding and gene expression observed in Fig. 5e cannot be fully separated from a potential normalization-dependent effect. Resolving this relationship will require orthogonal, stoichiometric measures of reader occupancy. Fourth, our analyses focused primarily on m6A-associated RBPs and m6A modifications. Other RBPs and RNA modifications, such as N1-methyladenosine (m1A), may also contribute to AD-associated gene regulation and warrant future investigation. Fourth, although generalized neuronal loss and coordinated activation of major cellular stress pathways were not detected under organoid culture conditions, more subtle or transient cellular stress responses cannot be completely excluded. In addition, our neuron-dominant experimental models do not fully capture the cellular complexity of the AD brain. Therefore, our findings should not be generalized to the full spectrum of AD-associated transcriptional alterations, particularly glial and immune programs. Fifth, our bulk RNA-seq-based computational framework may underrepresent regulatory changes occurring in less abundant cell types, including microglia. Single-nucleus RNA-seq data will provide improved resolution of cell-type-specific regulatory mechanisms and represent an important future direction for extending the DEcode-tree framework.

## Methods

### Participants

Participants came from ROS or MAP studies. The two studies began enrollment in 1994 and 1997, respectively [40]. ROS and MAP are conducted by the same team of investigators and share a large common core of measures, which allows combining the data for joint analyses. Participants enrolled without known dementia and agree to annual detailed clinical and cognitive evaluation, as well as brain donation after death. An Institutional Review Board of Rush University Medical Center approved all studies and participants gave written informed consent in accordance with the Declaration of Helsinki. As applicable, participants also sign an Anatomical Gift Act (AGA) for brain donation at death.

### Cognitive and clinical evaluations

AD diagnosis was based on criteria of the joint working group of the National Institute of Neurological and Communicative Disorders and Stroke and the Alzheimer’s Disease and Related Disorders Association (NINCDS/ADRDA) as previously reported [41]. Uniform structured cognitive and clinical evaluations are administered each year by examiners blinded to data from prior years. The cognitive battery contains a total of 21 cognitive performance tests. Briefly, 11 tests informed on diagnosis of AD dementia, mild cognitive impairment, and no cognitive impairment [41–43]. Further, 19 tests are used to construct a global composite measure of cognitive function [44]. The longitudinal rate of decline was computed for each participant using linear mixed models with adjustment for the effects of age, sex, and education, which estimate person-specific slopes. Additional models also controlled for common brain pathologies, resulting in person-specific residual decline as an estimate of resilience [45].

### Neuropathological examination

Tissue was dissected from 8 brain regions to quantify the burden of parenchymal deposition of beta-amyloid and the density of abnormally phosphorylated paired helical filament tau (PHFtau)-positive neurofibrillary tangles. Tissue sections (20 µm) were stained with antibodies against beta-amyloid protein and PHFtau protein, and quantified using image analysis and stereology, as previously described [45, 46]. In addition, the assessment captured cortical Lewy bodies, hippocampal sclerosis, TDP-43, macro- and micro-infarcts, as well as atherosclerosis and arteriolosclerosis [45, 46].

### RNA-seq gene expression

ROSMAP RNA-seq data of DLPFC regions were downloaded from the AMP-AD platform (syn3388564) and processed as previously described [12]. Briefly, paired-end RNA-Seq data were first aligned by STAR v2.6^48^ to the human reference genome (Release 27 GRCh38). Picard tools were used to assess the quality of the aligned bam files. Transcript raw counts were calculated by Kallisto (v0.46) [47]. Samples were excluded if the total mapped reads were less than 5 million. A total of 17,294 mRNAs were expressed in >50% of the sample with at least 10 counts in each sample. These genes were included in the downstream normalization process. First, the CQN (conditional quantile normalization) was applied to adjust a sequence bias from GC content and gene length [48]. Next, the adjusted gene counts matrix was converted to log2-CPM (counts per million), followed by quantile normalization using the voom function implemented in the limma R package [49]. Finally, a linear regression model was applied to remove major technical confounding factors, including post-mortem interval, sequencing batch, RQN (RNA quality number), total spliced reads reported by STAR aligner, and metrics reported by Picard and Kallisto.

### Mass spectrometry-based proteomics using isobaric TMT

We downloaded ROSMAP TMT proteomics data from frozen tissue of the DLPFC from the Synapse repository (syn30390636 and syn2580853). The detailed experimental procedure can be found in the description of the repository and the previous reports [50, 51]. Briefly, digested protein samples were labeled with isobaric TMT and fractionated using high-pH liquid chromatography. Fractions were then analyzed by LC-MS (liquid chromatography-mass spectrometry). The resulting MS spectra were searched against the Uniprot human protein database and quantified. The effects of the experimental batch, PMI, sex, and age at death on quantified protein abundance were regressed out using linear regression. Proteins with missing values in more than 50% of subjects were excluded. A total of 7,640 proteins in 596 persons passed the final QC. For replication analysis of protein-cognitive decline relationships, we utilized an independent TMT proteomics dataset (*n* = 288) from DLPFC regions generated through the AMP-AD project (syn51732482), encompassing multiple cohorts (ROSMAP, Minority Aging Research Study [52], and African American Clinical Core [53]) conducted by the Rush Alzheimer’s Disease Center with harmonized cognitive phenotyping and identical TMT protocols. In addition to the standard covariates, racial background was included as a covariate in the replication analysis.

### Transcriptome/proteome-wide association study

Associations between transcript or protein levels and continuous or categorical outcomes were tested using linear regression models implemented in the limma package (v3.50.3) in R [49]. Age at death, sex, and years of education were used as covariates to control for potential confounding factors. To test whether the differentially expressed genes reflect shifts in bulk cell-type composition (e.g., neuronal loss), we estimated per-sample cell-type proportions and re-fit the model adjusting for them. Proportions for the five major brain cell types (neurons, astrocytes, oligodendrocytes, microglia, endothelial cells) were estimated by reference-based deconvolution of the DLPFC bulk RNA-seq with the Digital Sorting Algorithm (DSA) [54], using markers from purified human cortical cell references [55].

### DEcode-tree model

We calculated the median transcript length and median intron length for each gene across all annotated transcripts to represent gene structures while accounting for transcript variability. To represent exon counts per gene, we computed a weighted average of exon numbers, with each transcript’s exon count weighted by its length, giving greater influence to longer transcripts. Furthermore, we calculated the weighted average GC content for the 3‘UTR, 5’ UTR, exon, and intron regions of each gene. For each region, the GC content of each transcript was weighted by its proportion of the total length of that region across all transcripts of the gene. For RNA and DNA features, we downloaded the genomic locations of binding sites for 172 RNA-binding proteins (RBPs) from POSTAR2 (as of October 2018), 217 microRNAs (miRNAs) from TargetScan Release 7.2, and 833 transcription factors (TFs) from the Gene Transcription Regulation Database (GTRD) (as of October 2018). Next, we mapped the binding sites of RBPs, miRNAs, and TFs to promoters and exons. Promoter regions for each gene were defined as the region spanning from 2,000 base pairs upstream to 1,000 base pairs downstream of the transcription start site (TSS). For cell-type-specific expression signatures, we utilized single-cell RNA sequencing (scRNA-seq) data from adult human brains [56]. Gene counts were quantified using Kallisto (v0.46) with annotations from GENCODE Release 27. Reads from the same cell type were aggregated to create pseudo-bulk datasets. We computed log2 counts per million (CPM) and calculated fold changes between cell types relative to the average expression across all cell types.

We trained predictive models using genomic and RNA features to estimate t-statistics for differential gene expression associated with cognitive decline and AD diagnosis. Extreme Gradient Boosting (XGBoost) regression models were implemented using the xgboost package (v1.7.7.1) within the R programming environment, interfaced with the mlr package (v2.19.1). The primary objective was to minimize the root mean square error (RMSE), with model performance evaluated based on RMSE and Pearson’s correlation coefficient between predicted and observed t-statistics. The model was configured with fixed parameters for the objective function, evaluation metric, number of boosting rounds, learning rate, booster type, number of processing threads, and regularization terms. Hyperparameter tuning was performed to optimize model performance. Adjusted hyperparameters included tree complexity parameters (maximum tree depth and minimum child weight), regularization strengths (L1 and L2 penalties), and subsampling ratios for both training instances and features. Hyperparameter optimization was conducted using systematic exploration within the mlr framework to identify the optimal model configuration based on the lowest RMSE and the highest correlation. To assess the model’s ability to predict differentially expressed genes (DEGs), we constructed receiver operating characteristic (ROC) curves by comparing predicted t-statistics to actual DEG status. The area under the ROC curve (AUC) was calculated to quantify classification performance. We estimated the AUC along with a 90% confidence interval from 100 bootstrap samples to account for variability in the model’s performance, providing a robust measure of predictive accuracy.

We employed SHapley Additive exPlanations (SHAP) to identify among the most influential predictive features. Predictive features were normalized by capping values at the 99th percentile and scaling to [0,1], regulatory binding site counts were normalized by maximum values with features having fewer than 30 positive observations excluded, and cell-type-specific expression values were scaled without centering. SHAP contribution values were computed for each feature using XGBoost’s built-in TreeSHAP implementation across five trained models, with SHAP values calculated on respective test sets using exact computation. Feature importance was ranked by averaging absolute SHAP values across all models and test samples. Enrichment analysis of m6A readers and interacting proteins of YTHDF1, YTHDF2, and YTHDF3 was conducted using the fgsea package (v1.20) with default parameter settings. RBPs were ranked by absolute SHAP importance scores, with gene sets defined from known m6A readers and protein-protein interactions. Statistical significance was assessed using false discovery rate (FDR < 0.05).

We analyzed relationships between predictive features using SHAP value correlations. Pairwise Pearson correlations were calculated between SHAP values across all features for each model separately, and the final correlation matrix was constructed by averaging correlations across models, retaining only features with complete correlation profiles. The correlation matrix was converted to a distance matrix using the transformation d = 1 - r, where r represents the correlation coefficient, and Uniform Manifold Approximation and Projection (UMAP) was applied to this distance matrix using the umap R package with parameters: n_neighbors = 15, min_dist = 0.5, and random_state = 42 for reproducibility.

### Protein co-abundance module analysis

To identify protein modules statistically, we employed the consensus clustering approach implemented in SpeakEasy (v1.0.0) [57]. We computed the composite metric for each module by averaging the normalized levels of all proteins within the module for each individual. To assess whether the protein modules are preserved in the transcriptome, we used the modulePreservation function from the WGCNA R package (v1.72) [58]. The network type was set to ‘signed,’ and all other parameters were kept at their default settings.

### Protein module impact assessment on DEGs

To assess the influence of m6A reader protein modules on cognitive decline-related DEGs, we computed two key metrics: the percentage of DEGs correlated with protein module levels and the percentage of remaining DEGs after accounting for module effects. First, DEGs associated with cognitive decline were identified from RNA-seq data from 549 samples with paired TMT proteomic measurements using the limma package, adjusting for covariates such as age at death, sex, and years of education. This analysis identified a total of 3,971 DEGs at an FDR of 5%. To examine the relationship between the DEGs and protein modules, we performed Pearson correlation analysis between the expression of each DEG and the eigenvalue (module summary) of each protein module. We then calculated the percentage of DEGs that showed significant correlations with module levels at an FDR of 5%. Following this, we conducted a regression analysis for each protein module to determine the DEG count after adjusting for the module’s effects. Module eigenvalues were included as covariates in the limma model to assess the contribution of each protein module individually. Finally, we calculated the reduction in the number of significant DEGs after adjusting for the effects of these modules, with significance assessed at an FDR of 5%. To assess whether a module’s contribution reflected its size or its overall strength of disease association, we additionally modeled the percentage of DEGs explained across all protein modules using a quasibinomial generalized linear model with module size (log₁₀) and disease-association strength as covariates. For each module, this model provided an expected percentage of DEGs explained under a size- and disease-association-matched null, and the deviation of the observed value from this expectation (standardized residual) quantified the module-specific contribution beyond these factors. The two m6A reader (YTHDF)-containing modules were analyzed as a pre-specified class; their excess DEG explanation was tested by adding a class indicator to the model, with significance assessed by a two-sided F-test.

### Cell line generation and maintenance

We used a commercially available Neural progenitor cell (NPC) line derived from XCL-1 iPSCs from Stem Cell Technologies (200–0620). This NPC line was generated from standard cortical differentiation, equivalent to early NPCs, based on previously published protocols from us and others [13–15, 59]. The NPCs were further matured into late NPCs by culturing and expanding them in STEMdiff Neural Progenitor Medium (StemCell Technologies, 05833) for 2 weeks. To establish stable Cas9 expression, early NPCs were transduced with lentiviruses carrying CRISPR Sp Cas9 pRCCH-CMV-Cas9-2A-Hygro virus at an MOI of 0.3 (Cellecta, SVC9-VS). After 48 hr, cells were selected with 100 μg/ml Hygromycin B (HygroB; Gibco, 10,687,010), cultured for 2 weeks to mature into late NPCs, and then banked and cryopreserved.

### Synthetic gRNA transfection

Synthetic guide RNAs targeting 8 m6A-relevant genes or 2 non-targeting (NT) controls were designed by and ordered from Synthego. Guide RNAs were provided as a lyophilized pool of two or three gRNAs, each targeting distinct sequences in exon 1 of the target gene. Each synthetic guide pool was resuspended in water to a 50 μM stock concentration. To perturb m6A-related RBPs, we first thawed and cultured the Cas9-expressing late NPCs for 6 days, then proceeded with transfections as described below. Cas9-expressing late NPCs were detached from dishes using Accutase, centrifuged at 300 ×g for 4 min, then resuspended in pre-warmed Neural Progenitor Medium (without HygroB) containing 20 μM Rho-kinase inhibitor (Y-27632; StemCell Tech 72304). Cells were counted and diluted to 1 × 10^6^ viable cells/ml. Meanwhile, gRNAs were diluted to 200 nM in sterile 1X TE buffer, pH 8.0 (220 μl each). In a 15-ml conical tube, 10 ml pre-warmed Opti-MEM media (Gibco; Thermo 31985070) was combined with 60 μl Lipofectamine RNAiMax (Thermo Fisher 13778150) and mixed well by inverting. For each KO target, 220 μl of diluted gRNA was combined with 800 μl Opti-MEM/RNAiMax mixture, pipette-mixed, and incubated at RT for at least 5 min but not more than 30 min. For transfection, the prepared cell suspension was plated onto pre-coated Matrigel plates (1 × 10^6^ cells in 1 ml for 6-well; 5 × 10^4^ cells in 50 μl for 96-well) and gRNA/Opti-MEM/RNAiMax mixture was immediately added to each well (950 μl for 6-well; 50 μl for 96-well), with a final concentration of 10 μM Y-27632 and 20 nM gRNA. Additional wells were subjected to mock transfection (using sterile TE buffer in place of gRNAs). Six hours after transfection, the media was removed and replaced with fresh Neural Progenitor Medium (without HygroB). Full media changes were performed again at 2-, 3-, and 4-days post-transfection (dpt) with fresh Neural Progenitor Medium (+100 μg/ml HygroB). At 5 dpt, genomic DNA (gDNA) was isolated from 96-well plates (see below), and cells in 6-well plates were passaged (using Accutase) to additional wells of matrigel-coated 6-well plates for further expansion, with daily media changes until 9 dpt. This protocol was repeated 2 times for a total of 3 biological replicates. The resulting cultures were then used for RNAseq and immunofluorescence staining. For staining, cells were fixed in 4% PFA for 15 min, blocked and permeabilized in 10% normal goat serum with 0.1% Triton X-100. The cells were stained with primary antibodies against MAP2 (Novus Biologicals NBP1-40606) and PAX6 (DSHB AB_528427), followed by species-specific Alexa Fluor-conjugated secondary antibodies (Thermo Fisher Scientific).

### Genomic DNA isolation and PCR

At 5 days post-transfection (dpt), media was aspirated and discarded from each well, then 100 μl of ice cold QuickExtract reagent (LGC Biosearch Technologies QE0905T) was added to each well. The plate was incubated at 65 °C for 6 min with 700 rpm shaking on a thermoshaker. Then 100 μl of lysate per well was transferred to a 96-well PCR plate. The plate was sealed, then incubated at 98 °C for 2 min followed by a 4 °C hold. Due to the high viscosity of isolated DNA, samples were diluted 4-fold in fresh QuickExtract reagent to enable accurate pipetting of low volumes. Undiluted and diluted gDNA were stored at −80 °C. PCR reactions were set up on ice using AmpliTaq Gold 360 polymerase (Applied Biosystems; Thermo Fisher 4398881) with the following components: 25 µl of 2X Master Mix, 0.5 µl of 10 mM forward primer, 0.5 µl of 10 mM reverse primer, 5 µl of GC enhancer, 15 µl of water, and 4 µl of gDNA, to a total volume of 50 µl. Primers were designed to amplify a 600–1200 bp fragment surrounding the Cas9 cleavage site, ensuring at least 200 bp between the primer binding site and any gRNA target region. Lysates from mock-transfected/WT or KO cells were used as templates. PCR cycling conditions were set as follows: enzyme activation at 95 °C for 10 seconds, followed by 40 cycles of denaturation at 95 °C for 25 seconds, annealing at 52 °C for 30 seconds (with the annealing temperature decreasing by 2 °C for each of the first four cycles, starting at 60°C), and extension at 72 °C for 1 minute. A final extension at 72 °C for 6 minutes was performed, with samples held at 4 °C.

### T7 endonuclease I (T7EI) assay

T7EI assay was performed according to the manufacturer’s instructions (NEB M0302L). Briefly, a 2.5% agarose gel containing SYBRSafe dye (Invitrogen S33102; diluted 1:10,000) was prepared. 12.5 μl of each PCR product was mixed with 1.4 μl NEB Buffer 2. The PCR products were then denatured at 95 °C for 5 min and re-annealed slowly (95–85 °C: −2°C/sec; 85–25 °C: −0.1°C/sec; 4 °C hold). 0.5 μl of T7 Endonuclease I (T7EI) was added to each sample, followed by 15 min incubation at 37 °C. 3 μl of 6X loading dye was then added to each T7-digested sample (WT and KO for each target), which were loaded to the agarose gel along with 100 bp ladder (Thermo Fisher SM0323) and run at 120 V for approximately 1 hour. UV images were captured using the Azure 300 Chemiluminescent Imaging System (Azure Biosystems). Band intensities were quantified using Fiji. Editing efficiency was estimated using the standard formula: 100 ×(1 – (1- fraction cleaved)^1/2^). The fraction cleaved was calculated by summing the intensities of the cleavage bands and dividing by the summed intensities of all bands (including cleaved and full-length). Intensities were normalized to the WT (unedited) sample so that the background intensity of cleavage bands = 0 for the WT.

### RNA isolation, library preparation, and sequencing

At 9 dpt, media was aspirated from two wells of a 6-well plate for each condition (8 KOs, 2 NT gRNAs). Cells were washed once with 1X DPBS, then 250 μl per well of DNA/RNA Shield buffer (Zymo R1100-250) was added. Plates were gently swirled until cells detached, and the solution pipette-mixed until homogeneous, then transferred to microcentrifuge tubes. RNA was extracted and concentrated using the RNA Clean & Concentrator (Zymo R1018) according to the manufacturer’s instructions. RNA was extracted similarly from 5 to 6 organoids per batch (*n* = 8 differentiations from 6 cell lines) across DMSO and YTHDF inhibitor-treated groups (*n* = 32 total samples; see “Cortical organoid culture and inhibitor treatment” below). For all KO and organoid samples, 500 ng total RNA was used to make RNA-seq libraries with the TruSeq Stranded Total RNA Library Prep kit (Illumina 20020599) following rRNA depletion (Illumina 20037135). Libraries were sequenced on an Illumina NovaSeq6000 to an average depth of 51 M reads per library. Sequencing data were obtained for the following KO samples: FMR1 (*n* = 8), FTO (*n* = 5), METTL14 (*n* = 8), METTL3 (*n* = 7), WTAP (*n* = 8), YTHDF1 (*n* = 8), YTHDF2 (*n* = 7), YTHDF3 (*n* = 7), and NT (*n* = 8).

### Bioinformatics analysis

To estimate the mutation rate introduced by the CRISPR system, we first aligned sequencing reads to the human reference genome (GRCh38, GENCODE Release 27) using STAR aligner [60] (v2.6). Aligned reads were then categorized based on their CIGAR strings into two groups: perfect matches and reads containing mismatches, insertions, deletions, or clipping. The mutation rate was calculated by determining the proportion of reads with any mutations relative to the total number of aligned reads. Gene expression quantification was performed using Kallisto (v0.46) with annotations from GENCODE Release 27. Genes expressed in more than 50% of samples with at least 10 counts in each sample were retained for differential expression analysis, resulting in a total of 16,378 genes. Data normalization was carried out using the voom method from the limma package [49], which models the mean-variance relationship in the log-counts. Differential expression between knockout (KO) and non-targeting (NT) guide RNAs (gRNAs) was tested using limma, controlling for sequencing batch, RNA preparation batch, and estimated library size.

### Western blot

At 9 dpt, media was aspirated from one well of a 6-well plate for each condition (8 KOs, 2 NT gRNAs, and mock-transfected/WT cells). Cells were washed once with 1X DPBS, then lysed in 250 μl in RIPA lysis buffer (Thermo 89,900) containing cOmplete^TM^ Protease Inhibitor Cocktail (Roche 11,697,498,001). Western blotting was performed using the Jess^TM^ Simple Western^TM^ (Biotechne 004–650) using the 12–230 kDa separation module (SM-W001). The Jess is an instrument enabling rapid/automated protein detection from low sample input. Lysate dilutions were optimized for each antibody and ranged from 1:3 to 1:9. Antibody dilutions were as follows: METTL3 1:20 (Abcam ab195352), METTL14 1:20 (Novus NBP1-81392), YTHDF2 1:20 (Abcam ab246514), YTHDF3 1:20 (Abcam ab220161), WTAP 1:20 (Abcam ab195380), FTO 1:40 (Abcam ab126605), Actin 1:50 (R&D MAB8929). Antibodies for YTHDF1 (ThermoFisher 17479–1-AP; Cell Signaling Technology 86463) and FMR1 (Biolegend 834701) were tested but did not produce a clean signal.

### Cortical organoid culture and inhibitor treatment

Human iPSC lines (BR62, BR63, BR01, BR21, BR22, BR28) were generated and characterized by the New York Stem Cell Foundation, and routinely maintained in mTeSR Plus medium (STEMCELL Technologies, #100–0276). The organoid differentiation was conducted based on published protocols with minor modifications [16]. In brief, iPSC were dissociated into single cells and aggregated at 10,000 cells/well of 96-well plate (S-Bio, MS-9096VZ) to form EBs in E6 medium (Fisher Scientific, A1516401) supplemented with Y-27632 (Tocris Bioscience, 1254) from day −1 to day 0. Neural induction (NIM phase) was initiated in E6 medium containing SB 431542 (10 μM) (Tocris Bioscience, #1614/10), LDN 193189 (100 nM) (Tocris Bioscience, #6053), and XAV 939 (2 μM) (Tocris Bioscience, #3748) from day 0 to day 4 to pattern EBs toward a cortical identity. From day 4 to day 10, XAV was withdrawn, while SB and LDN were continued. From day 10 to day 18, the medium was switched to neural differentiation medium (NDM phase) containing DMEM/F12, Neurobasal medium, N2 supplement, and B27 without vitamin A. From day 18 onward, 8 organoids were transferred to a single well of 6-well ultra-low attachment plate (Fisher Scientific, 07–200-601) on an orbital shaker and cultured in Neural Maturation Medium (NMM phase), supporting neuroepithelial rosette formation with twice-weekly media changes. The NMM medium consisted of DMEM/F12, Neural basal medium, *N*-2 supplement, B27 with vitamin A (Gibco, #17504–044), insulin (sigma, I9278), ascorbic acid (Cayman chemical, #16457), BDNF (ThermoFisher, #45002500UG), and dcAMP (Sigma, D0627).

Inhibitors of m6A readers were gifted from the laboratory of Dr Chuan He at the University of Chicago. The organoids (day 77) were treated with YTHDF1 inhibitor (SAC) at 20 μM, YTHDF2 inhibitor (DC-Y13-27) at 20 μM, combined SAC and DC-Y13-27 (both at 20 μM), and DMSO at 0.02% as the treatment control. 5 days later at day 82, organoids were collected for RNA extraction or fixed for 1 hour with 4% paraformaldehyde (Electron Microscopy Sciences, 15714-S) for immunohistochemistry.

For RNA-seq, organoids were generated from six iPSC lines derived from cognitively normal ROSMAP participants (BR62, BR63, BR01, BR21, BR22, BR28) across 1–2 independent differentiation batches, yielding *n* = 5–6 organoids per batch. For immunostaining, organoids were generated from two ROSMAP iPSC lines (BR62, BR63) across two independent differentiation batches, with 5–6 organoids per differentiation.

### Organoid IHC and image quantification

Fixed organoids were washed with 1x PBS, sunk in 30% Sucrose/1xPBS overnight, embedded in OCT, sectioned at 10 μm thickness, and mounted onto positive-charged glass slides, then stored at −80 °C until staining. The sections were permeabilized in 0.3% Triton X-100 in 1xPBS for 30 min, blocked in 5% BSA/1xPBS buffer for 1 hr, and immunostained in primary antibodies diluted in blocking buffer at 4 °C overnight. Primary antibodies included PAX6 (DSHB, AB_528427), FOXG1(Abcam, ab18259), TBR2 (Abcam, ab23345), TBR1 (Abcam, ab31940), CTIP2 (Abcam, ab18465), SATB2 (Abcam, ab51502), MAP2 (Abcam, ab5392), and 4G8 (Biolegend, #800712). After washing three times with wash buffer (0.15% Tween-20/1xPBS), the antibodies were detected by Alexa Fluor-488 goat anti-chicken secondary antibody (ThermoFisher, A-11039) and Alexa Fluor-546 goat anti-mouse secondary antibody (ThermoFisher, A-11003) at 1:500 dilution in blocking buffer for 1 h at room temperature. After washing five times with the washing buffer, the sections were further counterstained with Hoechst nuclear stain (ThermoFisher, H21486) for 5 min, and coverslipped with ProLong™ Gold Antifade Mountant (ThermoFisher, P36934).

The images were acquired by ORCA-Flash4.0 V3 Digital CMOS camera in Nikon Eclipse Ti2 epifluorescence microscope with objective lens of 20x. Acquisition settings were the same for all the slides.

ND2 images were next imported to ImageJ for quantification. Regions with folds in the tissue or otherwise obvious technical artifacts were excluded for downstream analysis. For each image channel (DAPI, 4G8, and MAP2), we applied a minimum intensity threshold to exclude low/background signal, which was consistent across all images. We then analyzed particle metrics for each channel (e.g. area and puncta size), and generated summary metrics (total area, average puncta size, number of puncta). Summary metrics for 4G8 were normalized by MAP2 area to account for organoid size and neuronal composition. Data was imported to R for plotting and statistics. We used a nested ANOVA with post-hoc Tukey tests to determine whether groups exhibited significant differences.

### m6A eCLIP

m6A-eCLIP was performed at Eclipse BioInnovations Inc, San Diego CA as described below. The procedure started by cryogrinding approximately 200 mg of human brain tissue and isolating total RNA using a Maxi Prep kit (Qiagen). The RNA quality and quantity was assessed by Agilent TapeStation. rRNA was depleted by coupling 80 μg of Eclipse-designed probes with 600 μL of Streptavidin beads (ThermoFisher) for 10 minutes at room temperature. 10 μg of total RNA and pre-coupled bead-probes were incubated separately at 72 °C for 30 seconds, then 67 °C for 90 seconds. Without removing samples from incubator, the total RNA sample was combined with pre-coupled bead-probes and incubated at 64 °C for 60 minutes. Following incubation, samples were magnetized, and the supernatant was reserved. RNA was cleaned using columns (Zymo) with a 200-nt size cut off. The mRNA quality and quantity was assessed by Agilent TapeStation. The mRNA was DNase-treated at 37 °C for 10 minutes then immediately sheared into 100–200 nt fragments by heating at 95 °C for 12 minutes in 1x Turbo DNase buffer (ThermoFisher). 4 μg of anti-m6A antibody (Eclipse Bio) was added, and samples were UV-C crosslinked using a UVP CL-1000 crosslinker at 2 rounds of 150 mJ/cm2 using 254 nm wavelength. The antibody-RNA complexes were then coupled overnight to protein G beads (CST). Following overnight coupling, library preparation (including adapter ligations, SDS-PAGE electrophoresis and nitrocellulose membrane transfer, reverse transcription, and PCR amplification) was performed as previously described for standard eCLIP [61], with size-selection performed cutting the 30–110 kDa region from the membrane (corresponding to RNA fragments crosslinked to antibody heavy and light chains). 5% of fragmented mRNA was run as an RNA-seq input control, starting with FastAP treatment as described [61]. The final library’s shape and yield was assessed by Agilent TapeStation.

### Reader protein eCLIP

eCLIP studies were performed by Eclipse Bioinnovations Inc (San Diego, CA) according to the published single-end seCLIP protocol as described with the following modifications. Approximately 300 mg of frozen human brain tissue was cryogrinded and crosslinked using a UVP CL-1000 crosslinker at 2 rounds of 400 mJoules/cm2 using 254 nm wavelength. Cryogrinded tissue was separated into 30 mg aliquots for each protein type and then stored until use at −80 °C. 30 mg of tissue was lysed using 1 mL of eCLIP lysis mix with a modified composition of 6 μL of Proteinase Inhibitor Cocktail and 20 μL of Murine RNase Inhibitor. Samples were then subjected to 2 rounds of sonication for 4 minutes with 30 second ON/OFF at 75% amplitude. Eclipse validated antibodies YTHDF1(ProteinTech: Cat No. 17479–1-AP), YTHDF2 (Aviva Systems Biology: Cat No. ARP67917_PO50), YTHDF3 (Abcam: Cat No. ab220161), and FMRP (Bethyl: Cat No. A305-200A) were then pre-coupled to Anti-Rabbit IgG Dynabeads (ThermoFisher 11203D), added to lysate, and incubated overnight at 4 °C. Prior to immunoprecipitation, 2% of the sample was taken as the paired input sample, with the remainder magnetically separated and washed with eCLIP high stringency wash buffers. Following overnight coupling, library preparation (including adapter ligations, SDS-PAGE electrophoresis and nitrocellulose membrane transfer, reverse transcription, and PCR amplification) was performed as previously described for standard eCLIP [61] with size-selection performed cutting the region from 65 kDa to 140 kDa for YTHDF1, 2 and 3 and from 65 kDa to 150 kDa for FMRP. RNA was visualized using Chemiluminescent Nucleic Acid Detection Module kit (ThermoFisher 89880).

### eCLIP analysis

The eCLIP cDNA adapter contains a sequence of 10 random nucleotides at the 5’ end. This random sequence serves as a unique molecular identifier (UMI) [62] after sequencing primers are ligated to the 3’ end of cDNA molecules. Therefore, eCLIP reads begin with the UMI and, in the first step of analysis, UMIs were pruned from read sequences using umi_tools (v1.1.0) [63]. UMI sequences were saved by incorporating them into the read names in the FASTQ files to be utilized in subsequent analysis steps. Next, 3’-adapters were trimmed from reads using cutadapt (v3.2) [64], and reads shorter than 18 bp in length were removed. Reads were mapped to the human genome (hg38) using STAR (v2.6.1d) [60]. PCR duplicates were removed using umi_tools (v1.1.0) by utilizing UMI sequences from the read names and mapping positions. Reads mapping to exons of GENCODE (Release 27) and not mapping to human repetitive elements (identified using the RepeatMasker track in the UCSC Genome Browser) were retained for further analysis. Peaks were identified within eCLIP samples using the peak caller CLIPper (v2.0.0) [65]. To reduce the computational time required to apply CLIPper to all samples, analysis was restricted to genes expressed in the input samples across m6A, YTHDF1, YTHDF2, YTHDF3, and FMR1 eCLIP experiments. Gene expression was quantified using featureCounts (v2.0.1) [66], and 11,633 genes with at least 10 counts in 50% of the samples were retained for CLIPper peak detection. For each peak, the enrichment of IP versus input was calculated using Fisher’s Exact Test based on a 2 × 2 contingency table comparing read counts within the peak region between IP and input samples. To control for multiple testing, Storey’s q-value method [67] was applied, and peaks with an FDR ≤ 5% were retained for each sample. Consensus peaks were defined as regions detected in at least 40 samples. These regions were then extended to include adjacent regions supported by at least 20 samples, and overlapping regions were merged to form the final consensus peak set. Read coverage for these consensus peaks was calculated across all samples using featureCounts (v2.0.1) with default parameters. For finer m6A site resolution, single nucleotide sites were also called. In this analysis, 5′ mismatches were trimmed from read alignments to remove potential artifacts. Single-nucleotide resolution crosslink sites were then called from the trimmed reads using PureCLIP v1.3.1 [68] utilizing input control data to identify sites enriched in the IP over the input. Enrichment of IP versus input at these sites was calculated using Fisher’s Exact Test as described above, and the q-value method [67] was applied to control for multiple testing, retaining sites with an FDR ≤ 5% in each sample. Consensus sites were defined as sites detected in at least 40 samples. Read counts overlapping these consensus sites were then quantified using bedtools (v2.30.0) [69].

### Peak locations along regional annotations

We visualized peak locations relative to transcript models using the Guitar package (v2.4.0) [70]. To ensure computational efficiency and maintain clarity in visualization, we randomly selected up to 10,000 peaks for analysis when the total number of peaks exceeded this threshold. Peak annotations were assigned based on their genomic regions, including promoters, exons, introns, and intergenic regions, to contextualize the binding sites within the transcript architecture.

### Motif analysis

We conducted de novo motif identification using HOMER [71] (v4.11) to search for enriched sequence motifs of length 5 base pairs (bp) within consensus peaks identified by CLIPper for each RNA-binding protein (RBP): YTHDF1, YTHDF2, YTHDF3, FMR1, and m6A eCLIP datasets. Given that de novo motif identification is a stochastic process and may yield variable results across different datasets, we aggregated all identified de novo motifs to create a comprehensive set for further analysis. To evaluate the enrichment of these aggregated motifs across all experimental conditions, we utilized the monaLisa package [72] (v1.0.0) in R. We selected the top two motifs from each eCLIP dataset based on statistical significance and assessed their enrichment across all antibodies using consistent parameters. To ensure a fair comparison between different datasets, we standardized the peak regions by extracting sequences of fixed length—100 bp centered around the peak summits—for all analyses.

### Differential peak analysis

Normalization of IP read counts for consensus peaks was conducted using DESeq2 [73] (v1.34.0), which models count data based on the negative binomial distribution. We adjusted for RNA Integrity Number (RIN), post-mortem interval (PMI), and sequencing batch effects in both IP read counts and gene counts in input samples. To account for IP efficiency and parental RNA expression levels, we followed the procedure outlined in a previous study [74]. Specifically, peaks within the top 1% of counts ranked by IP read counts were normalized by their corresponding parental gene counts. Correction factors for IP efficiency differences were then estimated by computing the median of each column after normalizing each row by the geometric mean. These correction factors were applied to the IP read counts to normalize for IP efficiency. To further account for differences in parental gene expression across samples, we calculated the ratio of gene expression differences against the mean expression across all samples for each gene. IP read counts were adjusted by dividing by this ratio for each gene, thereby normalizing for variations in parent gene expression levels. Differential peak analysis between AD and NCI samples was performed using DESeq2, with adjustments for sex, age at death, and years of education as covariates. False discovery rate (FDR) was estimated using Storey’s q-value method. Peaks were considered significantly differentially expressed if they met thresholds of *p* < 0.05 and FDR < 0.05.

### Predicting AD diagnosis from eCLIP and RNA-seq data

We performed elastic net regression using the glmnet package in R (version 4.1) to model the relationship between AD diagnosis and eCLIP as well as RNA-seq data from the DLPFC. Peak intensities from IP eCLIP data were normalized by the corresponding parental RNA expression levels to account for variability in RNA abundance. To reduce the number of variables in the elastic net model, we first identified co-expressed modules using the SpeakEasy algorithm with default settings (v1.0.0) [57]. For RNA expression data, we utilized the module definitions from our previous study [1]. Gene expressions were standardized by subtracting the mean and dividing by the standard deviation for each gene across all samples. An average module expression was then computed by taking the mean of the standardized expressions of all genes within each module. Model performance was assessed using a 5-fold cross-validation approach. Performance metrics included root mean square error (RMSE) and Pearson’s correlation coefficient between predicted and observed values. Hyperparameter tuning was conducted for the mixing parameter alpha, ranging from 0.1 to 1.0 in increments of 0.1, and the regularization parameter lambda, tested over a logarithmic scale from 1 × 10^−6^ to 100. The optimal model was selected based on the combination of alpha and lambda that minimized the RMSE and maximized the correlation between predicted and observed values. The model outputs the probability of a sample being AD-positive. We converted these probabilities to log odds and computed the area under the receiver operating characteristic curve (AUC-ROC) and the area under the precision-recall curve (AUC-PR) to evaluate model performance. All performance evaluations were conducted using test samples that were excluded from any model training processes. The PRROC package (v1.3.1) and the pROC package (v1.18.5) in R were used to compute these metrics.

## Electronic Supplementary Material

Below is the link to the electronic supplementary material.

Supplementary Material 1

Supplementary Material 2

Supplementary Material 3

## Data Availability

All data is available upon request through the RADC Resource Sharing Hub (http://www.radc.rush.edu) and the AMP-AD Knowledge Portal in accordance with the data usage agreement. The ROSMAP DLPFC RNA-seq, TMT proteomics, eCLIP, and organoid RNA-seq datasets are available for download from the AMP-AD Knowledge Portal (https://doi.org/10.7303/9618239). Gene expression data from CRISPR knockout experiments will be available on the Gene Expression Omnibus (GEO) under accession ID GSE286096. The AD Knowledge Portal is a platform for accessing data, analyses, and tools generated by the Accelerating Medicines Partnership (AMP-AD) Target Discovery Program and other National Institute on Aging (NIA)-supported programs to enable open-science practices and accelerate translational learning. The data, analyses, and tools are shared early in the research cycle without a publication embargo on secondary use. Data is available for general research use according to the following requirements for data access and data attribution (https://adknowledgeportal.org/DataAccess/Instructions). RNA-binding regions are available at POSTAR2 (http://postar.ncrnalab.org). Transcription factor binding regions are available at GTRD (https://gtrd.biouml.org).

## Materials availability

This study did not generate new unique reagents.

## Code availability

The DEcode-tree and eCLIP-seq analysis pipelines used in this study will be publicly available on GitHub (https://github.com/stasaki/DEcodeTree and https://github.com/stasaki/eCLIPseq-pipeline).
