# Supplementary material for: The YTHDF proteins modulate Alzheimer’s disease-associated brain gene signatures
Source: Mol Neurodegener. 2026 Sep 5;21:60. doi: 10.1186/s13024-026-00986-6 (PMC13625320; doi:10.1186/s13024-026-00986-6)
Supplement: Supplementary file 1 [file 13024_2026_986_MOESM2_ESM.docx]

**Supplementary Fig. 1: Cell composition analyses in the ROSMAP DLPFC cohort.** (**a**) Cell-type proportions estimated by RNA-seq deconvolution. Distribution of major brain cell types (astrocytes, endothelial cells, microglia, neurons, and oligodendrocytes) across cognitive diagnosis groups based on cell proportion estimates using the Digital Sorting Algorithm (DSA) on RNA-seq data from DLPFC tissue in the ROSMAP study (n=761). Groups include NCI (non-cognitive impairment, n=24), MCI (mild cognitive impairment, n=19), and Dementia (n=26). Boxes show the median and interquartile range (IQR), with whiskers extending to the most extreme value within 1.5×IQR of the hinge and outlying points plotted individually. For each cell type, cell proportions were compared across the three cognitive diagnosis groups using the Kruskal–Wallis rank sum test (an omnibus test of the three groups), and the resulting p-value is displayed above the corresponding boxes. (**b**) Comparison of differentially expressed genes before and after adjustment for estimated cell-type proportions. Venn diagram depicting the overlap of differentially expressed genes (DEGs) in Alzheimer's dementia with and without cell proportion adjustment. (**c**) Comparison of differential expression statistics before and after adjustment for estimated cell-type proportions.

**Supplementary Fig. 2: Model validation and application. (a)** DEcode-tree improves performance over the baseline DEcode model. Boxplot comparison of test set correlations for DEcode (baseline) versus DEcode-tree models, using the same set of features. Individual cross-validation fold results are shown as gray points. DEcode-tree shows a significant improvement over the baseline. **(b)** Reproducibility of differential gene expression statistics between data batches. The boxplot shows Pearson correlation coefficients between t-statistics from cognitive decline DEG analysis of RNA-seq batch 1 (full dataset) versus batch 2 (subsampled at various sizes). Each boxplot represents 10 iterations of random subsampling. Correlation increases with sample size but plateaus around 0.63, indicating an inherent ceiling for reproducibility between independent datasets due to biological variability and technical noise. **(c)** Percentages of differentially expressed genes (DEGs) associated with cognitive decline that are bound by RNA binding proteins (RBPs). Binding sites derived from the DEcode-tree features were compared with DEGs. Blue bars represent bound genes; gray represents unbound genes. **(d)** Reproducibility of model performance. Comparison of input type influence between RNA-seq batch 1 and batch 2. Variable importance was assessed using ablation analysis, repeated five times with different training and test gene sets. Comparison of variable importance scores between RNA-seq batch 1 and batch 2, based on SHAP scores. **(e)** Comparison between cognitive decline vs AD diagnosis. Comparison of input type influence between cognitive decline and AD diagnosis. Variable importance was assessed using ablation analysis, repeated five times with different training and test gene sets. Comparison of variable importance scores between cognitive decline and AD diagnosis, based on SHAP scores.

**Supplementary Fig. 3: Protein level validation. (a)** Forest plots showing meta-analysis of YTHDF proteins in AD brain samples. Meta-analysis was performed using the Stouffer method to combine p-values across independent batches. Z-scores with 95% confidence intervals are shown for YTHDF proteins. The meta-analysis combined data from Batch 1 (n=570) and newly generated Batch 2 (n=288) for a total of 858 samples. **(b)** Differential expression status of key RBPs and m6A regulators. RNA and protein level differences, associated with the rate of cognitive decline, were analyzed in 549 samples that had both RNA-seq and TMT proteomics data. An asterisk indicates statistical significance at nominal p < 0.05. (**c**) Module preservation of proteomics modules in transcriptome data. Module preservation was assessed using a pipeline implemented in the WGCNA package. Modules with a preservation statistic less than 2 were considered not preserved in RNA expression levels, including modules 58 and 43. **(d)** An example of how accounting for m43 affects the p-value distribution in DEG analysis for cognitive decline. (**e**) Observed versus expected percentage of DEGs explained across protein co-expression modules. DEGs explained (y-axis) versus the value expected under a matched null model relating DEG explanation to module size and strength of disease association (x-axis; quasibinomial generalized linear model, cbind(explained, remaining) ~ log₁₀(module size) + disease-association strength).

**Supplementary Fig. 4: Validation of m6A gene perturbation experiments.** **(a)** Brightfield image of cortical cells derived from Cas9-expressing NPCs used for CRISPR experiments. **(b)** Immunofluorescence image of cortical cells stained with the progenitor marker PAX6 and the neuronal marker MAP2. Scale bar: 100um in (a) and (b). **(c)** Gel image of T7 Endonuclease I assay result. Multiple cleavage products in the KO cells indicate genome editing at the intended site. Band intensities of full-length and cleavage products were quantified using Fiji, and gene editing efficiency was calculated using the following equation: 100 x (1 – (1- fraction cleaved)^1/2^) (see Methods). **(d)** Western blot results confirm the perturbation effects for m6A-associated RBPs. Whole-cell lysates from KO or NT control cells were subjected to western blot analysis using the Jess Simple Western platform. Digital gel images are shown for the 6 designated targets, probed for both Actin (red) and antibodies for the corresponding KO target (black). Actin-normalized intensities of KO targets are shown at the bottom. (**e**) Representative day-82 organoids for the BR21 line showing preserved cortical organization with progenitor and neuronal layers (scale bars = 100 microns). **(f)** IHC staining of day-82 organoids after five days treatment with DMSO control or the indicated YTHDF inhibitor(s). The red dashed boxes denote the area shown in Figure 3i (scale bars = 100 microns). **(g)** Quantification of MAP2 area normalized to DAPI area (ns: not significant).

**Supplementary Fig. 5: Differential expression of stress- and apoptosis-associated gene panels across DFi treatments.** Forest plot showing log₂ fold changes (relative to vehicle Control) for curated gene panels in organoid RNA-seq, comparing three treatments: DF1i (blue), DF2i (orange), and DF1i+DF2i (green). Each point is the estimated log₂ fold change for one gene under one treatment; horizontal bars denote 95% confidence intervals derived from the moderated standard error (±1.96 × SE). The vertical grey line marks no change (log₂FC = 0). Statistical significance was determined per treatment using Storey q-values; filled points indicate significant genes (q < 0.05) and open points indicate non-significant genes.

**Supplementary Fig. 6: eCLIP-seq quality control and peak detection.** UMAP plots displaying the similarity of peak locations detected from eCLIP-seq samples across all m6A readers and m6A. Each dot represents a sample, with colors denoting eCLIP-seq data for different reader proteins. The ratio of overlapping peaks between samples was calculated and visualized using UMAP with n_neighbors set to 30 and min_dist set to 0.8. **(a)** Results using all samples. **(b)** Results excluding library batch 2 of m6A eCLIP. **(c)** Summary of peak detection statistics.

**Supplementary Fig. 7: eCLIP-seq motif analysis and AD changes. (a)** De novo motif discovery was performed using HOMER, followed by motif enrichment comparison across different conditions using monaLisa. An asterisk indicates significance at a nominal p-value < 0.0001 using Fisher’s exact test. The number in each cell represents the percentage of peaks containing the motif. **(b)** Motif enrichment in m6A peaks comparing CLIPper vs pureCLIP. An asterisk indicates significance at a nominal p-value < 0.0001 using Fisher’s exact test. **(c)** Peak distribution comparison between CLIPper and pureCLIP, revealing a similar peak distribution of m6A. **(d)** de novo motif enrichment for peak groups. An asterisk indicates significance at a nominal p-value < 0.0001 using Fisher’s exact test. **(e)** Genes showing inverse relationships between expression and m6A reader binding in AD brain tissue. The heatmap displays Z-scores for genes showing significant differential expression (RNA row) and differential binding of m6A reader proteins (YTHDF1, YTHDF2, YTHDF3, FMR1, m6A rows) between AD and control samples. Columns represent individual genes selected for having significant differential binding across ≥4 m6A reader proteins with expression changes in the opposite direction. Red indicates positive Z-scores (upregulation/increased binding), blue indicates negative Z-scores (downregulation/decreased binding), and gray indicates non-significance.

**Supplementary Table 1**: Demographics for RNA-seq

**Supplementary Table 2**: Differential expression statistics

**Supplementary Table 3**: Feature importance for predicting CogDec DEGs

**Supplementary Table 4**: Demographics for TMT proteomics

**Supplementary Table 5**: Protein module assignments and within-module connectivity

**Supplementary Table 6**: Protein module association statistics, annotation, and influence on DEGs

**Supplementary Table 7**: DEG statistics for CRISPR-induced genes

**Supplementary Table 8**: Differentially expressed genes in DLPFC RNA-seq against various AD traits

**Supplementary Table 9**: DEG statistics for inhibitor-induced genes

**Supplementary Table 10**: Demographics for eCLIP-seq

**Supplementary Table 11**: eCLIP peak locations and annotations, and differential statistics

**Supplementary Table 12**: eCLIP peak differential statistics
