# Supplementary material for: The YTHDF proteins modulate Alzheimer’s disease-associated brain gene signatures
Source: Mol Neurodegener. 2026 Sep 5;21:60. doi: 10.1186/s13024-026-00986-6 (PMC13625320; doi:10.1186/s13024-026-00986-6)
Supplement: Supplementary file 2 [file 13024_2026_986_MOESM1_ESM.zip › Source_Data/Uncropped_gels_and_blots.docx]

**Source Data: Uncropped gels and blots (related to Supplementary Fig. 3c,d)**

Files are provided in Source_Data/ (T7/ and Jess/). For each gel/blot the uncropped image is shown first without annotation, then with the analyzed lanes outlined and labeled by target and condition (NT = non-targeting control; KO = knockout). Molecular-weight markers (kDa) are at left and lane numbers at the bottom of each image.

**Supplementary Fig. 3c — T7 endonuclease I assay (uncropped)**

The two images are the same agarose gel captured in two overlapping frames (the gel was wider than a single field of view); all lanes are represented across the pair. Multiple cleavage products in KO lanes indicate genome editing. Files: Source_Data/T7/T7_gel_1.tif and T7_gel_2.tif (original, unprocessed). For each gel the uncropped image is shown as acquired (light bands on a dark background); for clarity, the annotated panel below it is shown inverted (dark bands on a light background) with brightness/contrast adjusted so the outlined lanes are clearly visible.

*Source_Data/T7/T7_gel_1.tif — uncropped (shown as acquired)*

*T7_gel_1.tif — analyzed lanes outlined (shown inverted and contrast-adjusted)*

*Source_Data/T7/T7_gel_2.tif — uncropped (shown as acquired)*

*T7_gel_2.tif — analyzed lanes outlined (shown inverted and contrast-adjusted)*

**Supplementary Fig. 3d — Jess Simple Western (uncropped)**

Whole-cell lysates from KO or NT control cells were analyzed on the Jess Simple Western platform. Images are digital lane-view reconstructions; each capillary = one sample x one antibody. Red = Actin (total-protein control); black = the indicated target. Lanes are identified by the lane numbers at the bottom of each image. The figure uses the best exposure per target across three runs (one gel imaged at high, medium and low exposure, plus two additional gels). Files are in Source_Data/Jess/.

*Source_Data/Jess/2022-06-22_13-31-57_Jess_low-med_exp.png — 2022-06-22 (low-med) — FTO (lanes 9/10), ladder (lane 1) — uncropped*

*2022-06-22_13-31-57_Jess_low-med_exp.png — analyzed lanes outlined*

*Source_Data/Jess/2022-06-29_13-03-52_Jess_high.png — 2022-06-29 high exposure — METTL3 (16/17), WTAP (22/23) — uncropped*

*2022-06-29_13-03-52_Jess_high.png — analyzed lanes outlined*

*Source_Data/Jess/2022-06-29_13-03-52_Jess_medium.png — 2022-06-29 medium exposure — YTHDF3 (9/10) — uncropped*

*2022-06-29_13-03-52_Jess_medium.png — analyzed lanes outlined*

*Source_Data/Jess/2022-06-29_13-03-52_Jess_low.png — 2022-06-29 low exposure — YTHDF2 (13/14) — uncropped*

*2022-06-29_13-03-52_Jess_low.png — analyzed lanes outlined*

*Source_Data/Jess/2022-11-09_11-49-32_Jess_low.png — 2022-11-09 — METTL14 (2/3) — uncropped*

*2022-11-09_11-49-32_Jess_low.png — analyzed lanes outlined*

**Lane identity key (Supplementary Fig. 3d)**

| Panel | Source file (Source_Data/Jess/) | Lane ID |
| --- | --- | --- |
| Ladder | 2022-06-22_13-31-57_Jess_low-med_exp.png | 1 |
| METTL3 NT / KO | 2022-06-29_13-03-52_Jess_high.png | 16 / 17 |
| METTL14 NT / KO | 2022-11-09_11-49-32_Jess_low.png | 2 / 3 |
| WTAP NT / KO | 2022-06-29_13-03-52_Jess_high.png | 22 / 23 |
| FTO NT / KO | 2022-06-22_13-31-57_Jess_low-med_exp.png | 9 / 10 |
| YTHDF2 NT / KO | 2022-06-29_13-03-52_Jess_low.png | 13 / 14 |
| YTHDF3 NT / KO | 2022-06-29_13-03-52_Jess_medium.png | 9 / 10 |
